# Synergistic interfacial engineering of mesoporous magnetic metal oxide TiO_2_ nanocomposites for sustainable visible-light photocatalysis: Experimental insights and ML-based performance prediction

**DOI:** 10.1371/journal.pone.0348881

**Published:** 2026-06-02

**Authors:** Safdar Abbas Kazmi, Muhammad Saqib Khan, Muhammad Bilal, Muhammad Arshad, Rizwana Sarwar, Nfor Elvis Nfor, Muhammad Faisal Javed, S. Tasqeeruddin, Ajmal Khan, Ahson Jabbar Shaikh, Ahmed Al-Harrasi, Nadia Riaz

**Affiliations:** 1 Department of Environmental Sciences, COMSATS University Islamabad, Abbottabad Campus, Abbottabad, Pakistan; 2 Department of Environmental Sciences, Institute of Environmental Science and Engineering, School of Civil and Environmental Engineering (SCEE), National University of Sciences and Technology, Islamabad, Pakistan; 3 Department of Chemistry, COMSATS University Islamabad, Abbottabad Campus, Abbottabad, Pakistan; 4 Department of Civil Engineering Ghulam Ishaq Khan Institute of Engineering Sciences and Technology, Topi, Swabi, KPK, Pakistan; 5 Department of Pharmaceutical Chemistry, College of Pharmacy, King Khalid University, Abha, Saudi Arabia; 6 Natural and Medical Sciences Research Center, University of Nizwa, Birkat Al Mauz, Nizwa, Sultanate of Oman; 7 Department of Chemical and Biological Engineering, College of Engineering, Korea University, Seoul, Republic of Korea; Adama Science and Technology University, ETHIOPIA

## Abstract

This study investigates the structural, optical, morphological, magnetic, and photocatalytic properties of Fe_3_O_4_/TiO_2_ nanocomposites (FeT NCs), synthesized through a modified sol-gel method for the photodegradation of Reactive Yellow 145 (RY145). Characterization of FeT NCs (PL, XRD, FTIR, VSM, DRUV-Vis, DLS, Zeta potential, XPS, BET, SEM, TEM, TGA) revealed that Fe_3_O_4_ incorporation into TiO_2_ enhances charge separation, suppresses electron–hole recombination through Ti–O–Fe linkages, and improves photocatalytic efficiency. The calcined 0.025FeT3 exhibited high crystallinity with dominant anatase TiO_2_ and no rutile transition. SEM and TEM revealed a core–shell morphology with Fe_3_O_4_ cores encapsulated by TiO_2_, while aggregation was minimized by synthesis conditions. Optimal photocatalytic performance (84.51% % RY145 removal at neutral pH) was achieved using 1 mg mL^-1^ 0.025FeT3 following pseudo-first-order kinetics. The Langmuir–Hinshelwood model yielded rate and equilibrium constants of 2.80 mg.L^-1^ min^-1^ and 2.42 L mg^-1^, respectively. Mechanistic and scavenging experiments indicated that photogenerated holes and •OH radicals dominated the degradation process. The FeT catalyst maintained high stability over six cycles. Magnetic measurements showed soft magnetic behavior with low coercivity and remanence, favoring easy recovery. The reduced bandgap (2.62 eV) facilitated visible-light activation, while BET analysis confirmed a mesoporous structure with high surface area. XPS verified the oxidation states of Fe and Ti, and HPLC confirmed RY145 decomposition via azo bond cleavage and oxidation to carboxylic acids, demonstrating efficient and sustainable photocatalytic activity. 0.025FeT3 demonstrated efficient, stable, and magnetically retrievable photocatalytic activity under visible light, highlighting its potential for sustainable treatment of textile wastewater. To optimize the batch experimental data, a novel ML-driven predictive framework was tested to model and map the relationships between the selected optimization parameters (FeT contents, FeT dose, reaction time), to predict RY145 photodegradation efficiency, and to identify the optimal operating window for improved photocatalytic performance (using three regression measures R^2^, MAE, and RMSE). The CNN models outperformed with a predicted accuracy and R^2^ value of 0.91. Based on the results, ML-based evaluation outperformed manual optimization and traditional statistical methods, delivering a more efficient and reliable way for process optimization.

## 1. Introduction

Rapid industrialization and overexploitation of natural resources are irreversibly harming ecosystems, particularly aquatic areas such as lakes and rivers, which are heavily polluted. [1]. Millions of gallons of untreated wastewater are released every day from businesses such as textile and paper, damaging water bodies and emphasizing the critical need for effective, unfixed dye treatment. Azo dyes, which account for 70% of commercial dyes, are particularly dangerous because of their stability and resistance to deterioration. These dyes interfere with photosynthesis by blocking sunlight, and once they get into the food chain, they can cause cancer and genetic mutations, among other major health problems. [2]. A common example of a poisonous azo dye that can contaminate water and harm human skin and respiratory systems is Reactive Yellow 145 (RY 145), which is used in many textile mills, including those in Pakistan. It is probably stored and supplied for use in dyeing processes. [3]. Reactive Yellow 145 (RY145; CAS 93050-80-7) is a vinyl-sulfone anionic azo dye characterized by the molecular formula C_8_H_20_ClN_9_Na_4_O_16_S_5_ and a molecular weight of approximately 1026.25 g mol ^‒1^ [4]. Although no dependable data regarding its natural (untreated) environmental half-life in aerobic or anaerobic conditions exists, research on structurally related reactive azo dyes indicates persistence lasting over 100 days without treatment, highlighting their resistance in standard wastewater management systems. Toxicological evaluations have demonstrated notable acute and chronic effects; for instance, undegraded RY145 resulted in about 23.3% lethality in brine shrimp bioassays, while its biodegraded metabolites showed significantly reduced toxicity [5]. From a regulatory perspective, RY145 is included in the EU REACH/ECHA database (EC 279-408-8). With its high water solubility (≈ 80 g L ^‒1^ at 30 °C) and the presence of multiple sulfonate and azo groups, RY145 poses a risk of aquatic toxicity and prolonged persistence, requiring careful management and effective remediation techniques. Furthermore, as reported by the European Chemicals Agency (ECHA), RY145 may induce allergy or asthma symptoms or breathing difficulties when inhaled, thereby reinforcing the necessity for the development of efficient removal methods from industrial effluents. [6].

Addressing the dye-related pollution is crucial to safeguarding ecosystems and public health. Biosorption using fungal biomass [7], microbial degradation using *Klebsiella pneumoniae* [8], adsorption using teff straw-activated carbon [3] and hematite (α-Fe_2_O_3_) [9], conventional heating and microwave-based treatment [10] and advanced oxidation processes such as UV/H_2_O_2_ system [11], Ultraviolet-enhanced Ozonation (UV/O_3_) [12], electro-Fenton [13], chemical oxidation [14] and photocatalysis [15–17] are all common methods for RY145 dye removal from wastewater. Using semiconductor materials, photocatalysis has drawn interest because it can produce electron-hole pairs for simultaneous reduction and oxidation reactions. Titanium dioxide (TiO_2_) is a popular photocatalyst due to its high oxidizing capacity, non-toxic nature, and prolonged stability. Its wide bandgap (3.2 eV), however, restricts its practical applicability by limiting activation to UV light, fast electron-hole recombination, low quantum efficiency, and poor adsorption of non-polar pollutants. Additionally, nanoparticle aggregation limits active surface area, while challenging recovery and pH-dependent performance limit its efficiency. Photocorrosion and surface pollution are examples of stability concerns that impair long-term use. Strategies such as metal/non-metal doping, heterojunction creation, surface changes, and magnetic composites (e.g., Fe_3_O_4_/TiO_2_) are being investigated to improve photocatalytic efficiency and reusability [18].

Magnetic photocatalysts combine magnetic and catalytic capabilities, allowing for easier recovery using an external magnetic field, and resulting in increased recyclability. Magnetite (Fe_3_O_4_) is a popular photocatalyst because of its strong conductivity, superparamagnetism, huge surface area, and environmental compatibility. [19]. Combining Fe_3_O_4_ with TiO_2_ improves photocatalytic performance through charge carrier separation, electron acceptor/transfer channel, and efficient magnetic separation, making it an ideal candidate for practical applications. The Fe_3_O_4_/TiO_2_ nanocomposite (NC) has a core-shell structure, with Fe_3_O_4_ as the magnetic core and TiO_2_ as the outer photocatalytic layer. Fe_3_O_4_ improves the practical application of TiO_2_. The magnetic core facilitates recovery while simultaneously acting as an electron acceptor, enhancing charge separation and lowering recombination losses. Fe_3_O_4_/TiO_2_ NCs have superparamagnetic properties, large surface area, and conductivity, making them a sustainable solution for visible-light-driven photocatalysis and environmental cleanup. Relevant studies on Fe_3_O_4_-based photocatalytic NCs are summarized in S1 Table, emphasizing their multifaceted applications and promising attributes in addressing environmental challenges. VOSviewer bibliometric map (2015–2024) on Fe_3_O_4_/TiO_2_ composites and RY145 removal is presented as S2 Fig. in the supplementary materials.

Numerous studies have been conducted on the removal efficiency and reusability of Fe_3_O_4_/TiO_2_ NC for a variety of pollutants under different conditions of lighting. Xin et al. (2014) [20] highlighted a 74% final removal efficiency for Rhodamine B (RhB) under UV light after six reuse cycles, revealing the catalyst’s durability under repeated usage. After six cycles, Abbas et al. (2014) [21] achieved 90% final Methylene Blue (MB) elimination under UV-Vis light, demonstrating significant activity across a wider range of light wavelengths. Fe_3_O_4_/TiO_2_ NCs have demonstrated great initial performance, with complete degradation of Natural Red (NR) in a single cycle [22] and near-complete removal of Methyl Orange (MO) with 99.68% efficiency [23], highlighting the high initial performance of Fe_3_O_4_/TiO_2_ NCs. Multiple reuse cycles of Rhodamine 6G (R6G) and MB resulted in above 45% and 87.3% elimination, respectively, [24,25], indicating reasonable long-term performance. Furthermore, Gabelica et al. (2021) [19] showed a 91% final elimination of Ciprofloxacin (Ciprox) under UV-A light after three cycles, indicating the potential for antibiotic wastewater treatment. The cost-effectiveness of EDTA/Fe_3_O_4_@MBE NCs in heavy metal sequestration is demonstrated by their magnetic separability, better sequestration of Cd(II), Ni(II), and Zn(II), and regenerability of ≥89.07% after five recycles [26]. Variations in synthesis techniques, precursor types, calcination temperatures, reaction pH, initial pollutant concentrations, light sources, and degradation periods have all had a substantial impact on the photocatalytic efficacy of Fe_3_O_4_/TiO_2_ NC in previously published studies. Notably, many investigations have predominantly focused on UV or UV-Vis light sources, and high efficiencies frequently necessitated the use of acidic or basic media. In contrast, the current study demonstrates an innovative approach by reaching an initial RY145 removal effectiveness of 84.51%, which decreases to 68% after five cycles, under neutral pH circumstances and visible-light irradiation. This shows both the practical benefit of functioning in environmentally safe circumstances without requiring pH adjustment, as well as strong photocatalytic stability. The findings highlight the improved suitability of Fe_3_O_4_/TiO_2_ for treating wastewater in the real world, providing a blend of high degrading efficiency, reusability, and sustainable operation in mild and energy-efficient environments. MB dye was decolored by 58.18% when ZnO/C NPs were used [27], whereas green Fe_3_O_4_ NPs synthesized from Citrus limetta peels biowaste extract efficiently sequestered 98.24% of Cd (II) from electroplating effluent up to five cycles [28]. Using Nd-TiO_2_ for real textile wastewater (at 1:9 dilution) treatment under UV and solar light, maximum degradation reported was 96% and 91% [29]. The effect of the primary value of pH on photocatalytic removal of amoxicillin using Fe-TiO_2_@Fe_3_O_4_ (primary concentration of amoxicillin: 40 mg/L, reaction time: 75 min at pH values 3–11), with maximal productivity of degradation attained at a pH of 7 and an NP level of 600 mg und UV light irradiation [30].

This study focuses on a mesoporous Fe_3_O_4_/TiO_2_ NCs designed to improve photocatalytic performance, addressing limitations of traditional binary composites that typically use simple physical mixing (S1 Table). The novel structure enhances charge transfer and surface reaction kinetics, increases surface area, and improves mass transport of reactants. Importantly, the nanocomposite is effective under visible light at near-neutral pH, making it suitable for real wastewater treatment. Additionally, Fe_3_O_4_ facilitates magnetic recovery and reduces electron-hole recombination, thereby enhancing overall efficiency. This work integrates interfacial engineering, visible-light responsiveness, and practical usability in photocatalytic systems. This study engineered and optimized visible-light-active Fe_3_O_4_/TiO_2_ NCs with improved charge separation, mesoporosity, and magnetic recoverability for the effective breakdown of RY145 dye under visible light in neutral pH conditions. The problem addressed stems from the inherent drawbacks of pure TiO_2_, which limit its efficiency in practical wastewater treatment. These limitations include its broad bandgap (3.2 eV), fast electron–hole recombination and limited visible-light response. To overcome these constraints, this research introduces a magnetic heterojunction strategy by integrating a low-loading Fe_3_O_4_ shell (~0.025 mol%) into the TiO_2_, synthesized via the sol-gel technique and optimized via controlled calcination (≈300 °C as the best). This study also integrated an innovative machine-learning (ML)-driven predictive framework to improve the reliability and interpretability of experimental results acquired through batch photocatalytic experiments. The nonlinear and multivariable interactions between important operating factors (synthesis and reaction) that collectively influence photocatalytic performance, such as FeT contents (mol%), (b) FeT dose 1 mg/ mL, and reaction time (RT in minutes), are frequently difficult for traditional optimization tools to capture. By identifying intricate patterns in the information and facilitating the accurate prediction of degradation outcomes under various circumstances, machine learning (ML) offers an effective alternative. This study trained and evaluated four deep-learning models utilizing three regression performance metrics (R², MAE, and RMSE) to forecast and enhance RY145 removal performance. The incorporation of ML not only allowed for the identification of the most effective photocatalytic performance window but also competed with manual optimization and conventional statistical approaches regarding durability and computational effectiveness. Furthermore, the use of machine learning directly supports the research aims by expediting parameter optimization, reducing experimental effort, and providing a data-driven foundation for creating high-performance photocatalysts. The engineering hypothesis states that minimal Fe_3_O_4_ concentration combined with controlled thermal treatment forms a stable type-II Fe_3_O_4_/TiO_2_ heterojunction with adequate interfacial Ti-O-Fe coupling, providing efficient charge carrier separation and retaining a mesoporous structure and soft magnetic characteristics. With competitive electrical energy per order (energy cost ($/m³) or EEO ≈ 0.5 kWh/m³ * 0.09 $/kWh), efficient visible-light-driven degradation of RY145 at neutral pH, rapid magnetic recovery (> 90% within 30s), and minimal metal leaching (< 0.2 mg·L^‒1^), this configuration demonstrates both environmental compatibility and scalability for actual wastewater treatment applications.

## 2. Materials

All the chemicals used in this study, along with the estimated cost and lab-scale cost estimation of the synthesized composite, are listed in S3 Table:

### 2.1. Synthesis of Nanoparticles (NPs)

#### 2.1.1. Synthesis of TiO_2_.

TiO_2_ photocatalysts were synthesized via a modified sol-gel method. [31]. In a typical sol-gel method, titanium tetraisopropoxide (TTIP) (10 ml) was first mixed with absolute ethanol (40 ml) to form solution A. Meanwhile, solution B was prepared by combining deionized distilled water (5 ml) and acetic acid (5 ml) in absolute ethanol (40 ml). Solution B was then added dropwise (introduced at a controlled rate) to solution A while maintaining vigorous stirring to ensure thorough mixing. The mixture was continuously stirred until a gel-like consistency was achieved. Once the gel was formed, it was allowed to age for 24 hours under ambient conditions to promote further polymerization. Following this aging period, the gel was dried in an oven at 70 °C for 24 hours. After drying, the resulting material was ground into a fine powder and stored for future use.

#### 2.1.2. Synthesis of Fe_3_O_4_.

The co-precipitation synthesis of substituted ferrite nanoparticles involved critical reaction parameters like temperature, pH, and initial molar concentration. This method [32] included co-precipitating (NH_4_)_2_Fe(SO_4_)_2_ and FeCl_3_ in an alkaline medium, maintaining the mixture at 80 °C. Magnetite was formed through the conversion of metal salts to hydroxides and subsequent transformation into ferrites in a boiling NaOH (0.5 M) solution under continuous stirring for 10 seconds. Fe_3_O_4_ nanoparticles were finally obtained following washing with distilled water and drying.

### 2.2. Synthesis of Fe_3_O_4_/TiO_2_

One of the most widely employed and effective chemical precipitation methods [33] was used to synthesize Fe_3_O_4_ NCs as briefed in section 2.1.2. To synthesize Fe_3_O_4_/TiO_2_ (FeT) the different quantities having corresponding mole% [0.01, 0.025. 0.05, 0.1, 0.2, and 0.5] of already synthesized Fe_3_O_4_ was dispersed in 30 mL of isopropyl alcohol and sonicated for 30 minutes. 37 mL TTIP dissolved in 60 mL ethanol plus acetic acid was carefully added to the mixture and stirred for two hours. The resulting product, as gel was dried for 24 hours at 100 °C in an oven. After grinding, the resultant powder sample was stored in an air-tight container till further use.

### 2.3. Characterization

A variety of characterization tools were used to characterize the photocatalyst to have a better understanding of its physicochemical features and to obtain information that may be useful in improving the connection between the physicochemical properties and photocatalytic performance. The best-performing photocatalysts were selected for characterization to understand their photoluminescence properties, phases, and crystallite size, surface morphology, elemental composition, surface chemistry and bandgap estimation. The photoluminescence spectrum (PL) was obtained using a Fluorescent Spectrophotometer (RF-6000, Shimadzu, Japan). Crystallite phases and size were analyzed using an X-ray diffractometer (XRD) (JDX-3532, JEOL, Japan). Bandgap estimation was achieved by diffuse reflectance spectroscopy (DRS-UV-2600i, Kyoto, Japan), crystalline phase identification, and functional groups were determined by Fourier-transform infrared spectroscopy (FTIR-Alpha Bruker, Karlsruhe, Germany). The absorption spectrum and bandgap estimation data were obtained using Lambda 365 + Perkin Elmer UV Vis Spectrophotometer. Surface morphology and elemental composition were evaluated using an atomic force microscope (JEOL JSPM-5200 Tokyo, Japan), scanning electron microscopy (SEM) with energy dispersive spectroscopy (EDS) using a JEOL JSM-6510LA, Tokyo, Japan, and transmission electron microscopy (TEM) using JEM 1400. Dynamic Light Scattering (DLS) was performed to determine the hydrodynamic size distribution, and Zeta Potential analysis was conducted to evaluate the surface charge of the nanocomposites. The magnetic hysteresis behavior of photocatalysts was analyzed using Vibrating Sample Magnetometry (VSM). Thermogravimetric analysis (TGA) of the uncalcined 0.025FeT nanocomposites was conducted using a PerkinElmer Pyris instrument to assess its thermal stability and decomposition behavior with rising temperature. A 5.300 mg sample was heated from 35°C to 800°C at a constant rate of 10°C/min under an air atmosphere

### 2.4. Photocatalytic degradation studies of RY145 dye

Photocatalytic degradation experiments using RY145 as a model pollutant were conducted under the visible light source of a 500 W Halogen lamp (Hi Luminar-Germany) placed at a distance of 25 cm (30798 lux light intensity), ambient temperature, 50 mg L^-1^ of RY145 concentration, 6.2 pH, and 1 g L^-1^ photocatalyst dose. Initially, the synthesized FeT photocatalyst was dispersed in distilled water through ultrasonication followed by the addition of a required amount of RY145 dye. Before the photocatalytic degradation experiments, the suspension was magnetically stirred for 30 min in the dark followed by illumination for 60 min under the mentioned light source. Samples were collected at a pre-determined interval for RY145 adsorption (in dark) and discoloration (in light). A UV-visible spectrophotometer (PG instruments T80^+^, Lutterworth, UK) was used to investigate the RY145 discoloration at the specific wavelength (419 nm). The calibration curve (shown in S4 Fig.) was developed with standard solutions of different RY145 concentrations. The suspended particles of photocatalysts were removed through an external magnet and centrifugation (to ensure complete removal of suspended particles) each time before the absorbance measurement. RY145 discoloration (%) was determined using Equation (1).


$$\begin{array}{c} RY\text{1}\text{4}\text{5}\;Discoloration(\%)=\left(\frac{{\text{C}}_{\text{0}}-{\text{C}}_{\text{t}}}{{\text{C}}_{\text{0}}}\right)\text{x100} \end{array}$$ (1)

Where C_0_ and C_t_ represent the RY145 concentration, respectively, before and after irradiation at time *t*. Moreover, the effect of different reaction parameters including the contact time, pH, FeT dose, and initial dye concentration, on RY145 discoloration was investigated with the same experimental setup and procedure explained above. Equation 2 was utilized to calculate the percentage of Chemical Oxygen Demand (COD) removal, to assess the degree of mineralization. This involved analyzing the initial and final concentrations of the samples collected after reaction studies, complemented by HPLC analysis to investigate the resulting reaction by-products and ensure a comprehensive understanding of the degradation process.


$$\begin{array}{c} \text{Dye Mineralization }(\%)=\left(\frac{COD_{\circ }-COD_{f}}{COD_{\circ }}\right)\times \text{100}\;\;\;\; \end{array}$$ (2)

Where: COD₀ is the initial Dye COD (ppm) and CODf is the Dye concentration at different intervals of time for the period of light irradiation.

The photodegradation of RY145 and formation of intermediate by-products following treatment with the FeT NCs were evaluated on a Shimadzu (Kyoto, Japan) Nexera UHPLC system equipped with a photodiode array (PDA). The separation was done on a reversed-phase C18 analytical column at ~30°C. The stationery and mobile phases included acetonitrile and methanol with an injection volume of 10 µL, and the flow rate was set at 0.5 mL/min. Each reaction sample was centrifuged for 10 minutes at 10,000 rpm before being filtered through a 0.22 µm PTFE syringe filter for the removal of any suspended particles or NCs. The chromatograms of treated samples were compared to those of the untreated RY145 solution to assess the removal of the parent dye peak and the emergence of additional peaks corresponding to potential degradation intermediates. The intermediates, such as aromatic amines, hydroxylated aromatic compounds, and low-molecular-weight carboxylic acids, were provisionally assigned based on retention times and peak intensities.

### 2.5. Active species trapping studies

Studies on active species trapping were performed to explore the photocatalytic mechanism of FeT using specific scavengers including Sodium chloride (NaCl), Sodium sulfate (Na_2_SO_4_), Calcium chloride (CaCl_2_), and isopropyl alcohol (IPA). Four scavengers were added to the reaction system individually: IPA and NaCl were utilized as selective hydroxyl radical (•OH) scavengers, Na_2_SO_4_ as a hole (h^+^) scavenger, and isopropyl alcohol (IPA). In the case of NaCl, Cl^‒^ ions react quickly with •OH to reduce its oxidative contribution. Na_2_SO_4_ was used as a hole (h^+^) scavenger, competing with pollutants for photogenerated holes, while CaCl_2_ quenched both •OH and superoxide radicals (•O_2_^‒^) via chloride-driven radical suppression and ionic strength effects. The inhibition of RY145 degradation seen after the addition of each scavenger allowed the identification of the primary reactive species controlling the photocatalytic pathway. One reaction was carried out without the addition of any scavenger (NSA). These investigations assist in identifying the active species involved in the photocatalytic degradation of pollutants by observing the changes in degradation efficiency with each scavenger. The findings elucidate the functions of different reactive species, such as •OH, •O_2_^‒^ and h^+^ in the photocatalytic discoloration process of RY145, and provide valuable insight into the mechanistic pathways governing the photocatalytic discoloration of RY145.

### 2.6. Photocatalytic kinetics

Langmuir, Freundlich and Langmuir-Hinshelwood (L-H) model [34] was employed to elaborate on the rate of the photocatalytic decoloration of RY145 dye over time. The L-H model for the photocatalytic system can be explained as:


$$\begin{array}{c} \frac{\text{1}}{r_{\text{0}}}=\frac{\text{1}}{k_{c}}+\frac{\text{1}}{k_{c}K_{LH}}.\frac{\text{1}}{[RY\text{1}\text{4}\text{5}{]}_{e}} \end{array}$$ (3)

The dependency of 1/r_0_ for the corresponding 1/[RY145]_e_ concentration values of RY145 can be translated by Equation (3). In comparison, the k_c_ and K_LH_ values demonstrate the effect of the RY145 concentration on the equilibrium constant.

### 2.7. Recycling studies

Recycling studies were performed using photocatalysts with dominant performance, i.e., 0.025FeT3 NCs. The used photocatalyst was washed with deionized water, centrifuged and dried at 80 °C overnight. After drying, the recovered photocatalyst was reused under the same experimental conditions as described in section 2.4 and decoloration (%) was determined using equation (1).

### 2.8. Machine learning models

To forecast RY145 removal efficiency and capture nonlinear interactions among important experimental variables, such as FeT contents (mol%), (b) FeT dose 1 mg/ mL, reaction time (RT in minutes), four machine-learning models (ANN, FNN, CNN, and RNN) were created using TensorFlow/Keras. Direct input-output mappings were learned using ANN and FNN as multilayer perceptrons, hierarchical feature patterns were extracted using CNN via 1D convolutions, and time-dependent degradation behavior was modeled using RNN (LSTM/GRU). All models were trained and optimized in Google Colab using hyperparameter tuning (S4 Table), with the output target set to RY145 dye removal (%). Three statistical indices (R², MAE, and RMSE) were used to evaluate model performance, including variance explanation, absolute error, and squared error. This integrated ML methodology allowed for more accurate predictions of photocatalytic efficiency, identification of optimal parameter ranges, and a better understanding of complicated reaction variable interactions than traditional statistical approaches. Further details of the model used are explained in the following sections.

#### 2.8.1. Artificial neural network (ANN).

This is a general name for interconnected neurons/layers that learns nonlinear input → output mapping used for predicting pollutant removal, degradation rate, or percentage removal from experimental variables (pH, catalyst dose, irradiation time, and pollutant concentration, etc.) [35].


$$\begin{array}{c} \text{Y }=\;f(𝐖𝐱\;+\;𝐛) \end{array}$$

$$\begin{array}{c} \text{For an L}-\text{layer MLP }(\text{ANN}): \end{array}$$

$$\begin{array}{c} {\text{a}}^{\left(o\right)}\;=\text{ x},\;{\text{z}}^{\left(l\right)}\;={\text{W}}^{\left(l\right)}\;{\text{a}}^{\left(\text{l}-\text{1}\right)}\;+\;{\text{b}}^{\left(l\right)}\;,\;a^{\left(l\right)}\;=\sigma ({\text{z}}^{\left(l\right)})\; \end{array}$$

$$\begin{array}{c} \text{Final output }\hat{y}\;=\;a^{\left(l\right)} \end{array}$$ (4)

Where: x is the input feature vector (pH catalyst dose, pollutant concentration reaction time, etc,) l is the layer index, W^(l)^ is the weight matrix connecting layer l – 1 to layer l, b is the bias for vector added at layer l, z^(l)^ is the linear pre-active output of layer l, a^(l)^ activated (nonlinear) output of layer l, a^(o)^ input vector (same as x), σ(.) is the activation function, ŷ is the prediction output (% degradation)

#### 2.8.2. Feed-Forward Neural Network (FNN).

This is a subtype of ANN where information flows only forward (no recurrence). Most photocatalysis ANN applications actually FNNs/MLPs (Multi-layer Perception), simple, fast to train, effective for static experiment input → output prediction [36]. Same mathematical application as in ANN, with Loss commonly mean square error (MES) for regression.


$$\begin{array}{c} \text{Y }=\;f(𝐖𝐱\;+\;𝐛) \end{array}$$

$$\begin{array}{c} \text{For an L}-\text{layer MLP }(\text{ANN}): \end{array}$$

$$\begin{array}{c} {\text{A}}^{\left(o\right)}\;=\text{ x},\;{\text{z}}^{\left(l\right)}\;={\text{W}}^{\left(l\right)}\;{\text{a}}^{\left(\text{l}-\text{1}\right)}\;+\;{\text{b}}^{\left(l\right)}\;,\;a^{\left(l\right)}\;=\sigma ({\text{z}}^{\left(l\right)}) \end{array}$$

$$\begin{array}{c} \text{Final output }\hat{y}\;=\;a^{\left(L\right)} \end{array}$$ (5)

Where: x is the input feature vector (pH catalyst dose, pollutant concentration reaction time, etc,) l is the layer index, W^(l)^ is the weight matrix connecting layer l – 1 to layer l, b is the bias for vector added at layer l, z^(l)^ is the linear pre-active output of layer l, a^(l)^ activated (nonlinear) output of layer l, a^(o)^ input vector (same as x), σ(.) is the activation function, ŷ is the prediction output (% degradation)

#### 2.8.3. Convolutional Neural Network (CNN).

This is a network architecture with convolutional layers that teach local patterns (filters). In photocatalysis, it is used less for plain tabular data and more for structure-aware inputs or when augmenting data. Some studies used CNN to predict active sites. CNN can outperform ANN when spatial or structural input is important [37].


$$\begin{array}{c} \text{1D}:\;(x\;*\;w)\;[t]=\sum_{\text{i}}\;x\;[t\;=\;i]w[i] \end{array}$$ (6)

Where: x, X is the signal (1D) or features matrices, w, is the convolutional kernel/filter (learnable weights), t, is the output indices, *i* are the filter element indices (x*w), convolutional result (feature predictions or output),

#### 2.8.4. Recurrent Neural Network (RNN).

This includes Long Short-Term Memory Network and Gate Recurrent Unit (LSTM/GRU) variants designed for sequential/temporal data (time series). RNN models time dependencies in photodegradation experiments taken as time series sensor streams, or process dynamics. LSTM/GRU alleviates vanishing gradients for longer sequences. Used when temporal evolution matters (kinetics, reactor monitoring) [38].


$$h_{t}=\;\phi ({\text{W}}_{x}{\text{x}}_{t}={\text{W}}_{h}{\text{h}}_{t-\text{1}}+b)$$

$$\begin{array}{c} {\hat{y}}_{t}\text{ g }({\text{W}}_{y}{\text{h}}_{t}=\text{c}) \end{array}$$ (7)

Where: x_t_ is the input at time t, h_t_ is the hidden state at time t (memory of the network), h_t-1_ is the hidden states from previous time step, W_x_ is the input-to-hidden weight matrix, W_h_ is the hidden-to-hidden recurrent weight matrix, W_y_ hidden-to-output weight matrix, b is the bias for the hidden layer, c is the bias for output layer, g(.) is the output activation (usually linear for regression), t is the time index, ŷ _t_ predicted output at time t.

### 2.9. Model development and performance evaluation

The TensorFlow/keras package is designed to develop and refine the selected deep learning model algorithms. For the experiments, Google Collaborative Notebook Python (Google Colab), hyperparameter optimization was carried out on the data from the reaction parameters chosen (supplementary information): the FeT concentration (mol%, IFeC), reaction time (RT in minutes), and FeT dose (AD as g/L), as the input variables, while the dye removal percentage served as the target or outputs. The network architecture for the 4 models is presented in S5 Fig.

#### 2.9.1. ANN Architecture.

The ANN architectural model, which uses feedforward with 2 hidden layers to understand the complex relationship between IFeC, RT, and AD in relation to dye removal efficiency (S5-a Fig.). Applying the model in photodegradation studies helps excel at capturing how diverse applied parameters work together in breaking down pollutants. ANN has a knack for generalizing experimental data and is particularly effective in predicting the optimal conditions to achieve maximum dye degradation, particularly when there is a lot of variability in the experiments [39,40]. Its flexibility has made it a go-to choice for modeling heterogeneous photocatalytic systems.

#### 2.9.2. FNN architecture.

A subtype of ANN featuring multiple layers in a feedforward network system and provides a robust learning capacity that is perfect for tackling complex photodegradation processes with multiple reaction pathways (S5-b Fig.). FNN have proven effective in predicting the degradation rates of dye like methylene blue and Rhodamine Blue under different photocatalytic conditions [35]. The deep model can effectively simulate feedforward multi-step degradation kinetics and finetune treatment parameters.

#### 2.9.3. CNN Architecture.

This model shows great effectiveness in modeling photocatalytic degradation, particularly paired with spectral or image-based inputs [41] using convolutional layers (S5-c Fig.). Through the hidden interaction of layers, CNN can be utilized to pinpoint complex features like IFeC, RT, and AD on the kinetics of odd dye breakdown.

#### 2.9.4. RNN architecture.

The model is grafted to capture the nuances of time, making it perfect for modeling the ever-changing processes of photodegradation (S5-d Fig.). It has been reported to forecast degradation curves and calculate reaction constants in UV/TiO_2_ systems and Fenton-like processes [42], providing valuable insights into reaction dynamics that go beyond mere static predictions.

### 2.10. Statistical performance metric indicator

ML model development entails a systematic workflow that includes data processing, model training, hyperparameter optimization, and validation, with each model algorithm trained on an experimental dataset to capture nonlinear and complex relationships and project target variables to varying degrees of accuracy. Four statistical indicators are used in this work to assess the performance of the deep learning models: root mean square error (RMSE), mean absolute error (MAE), and coefficient of determination (R^2^). Each of these metrics provides completeness within R^2^ to assess the proportion of variation explained and predictive skill, whereas MAE and RMSE measure absolute and square deviation. The standard for these statistical indicators is presented in Table 1.

**Table 1:** Statistical indicators and acceptable ranges of model performance.

Metrics	Formula	Acceptable standard range	Reference
R^2^	${\text{R}}^{\text{2}}=\text{1}-\frac{\sum (Yi-\hat{\text{Y}}i{)}^{\text{2}}}{\sum {\left(Yi-Yi\right)}^{\text{2}}}$	0 ≤ R^2^ ≤ 1	[43,44]
MAE	$MAE=\frac{\text{1}}{n}\sum_{i=\text{1}}^{n}|yi-\hat{y}i|$	<10% is good	[45]
RMSE	$RSME=\sqrt{\frac{\text{1}}{n}}\sum (Yi-\hat{\text{Y}}i{)}^{\text{2}}$	Often accepted if < 10% of the mean (approaching 0 is good for the model)	[46]

Yi = predicted or actual values for i^th^ data point, ŷi = predicted model values for i^th^ data point, ȳi = mean of observed values, n = total number of data points, Σ = summation of all observations i = 1….. n

## 3. Results and discussion

### 3.1. Characterization

#### 3.1.1. PL spectroscopy.

**Fig 1:**
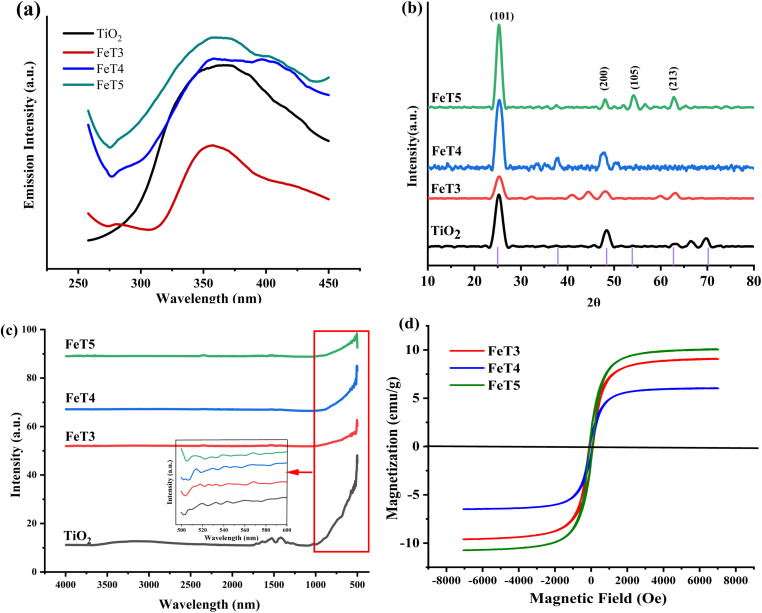
(a) PL emission spectra UV–vis spectra, (b) XRD plot, (c) FTIR spectra, (d) Magnetic hysteresis curves of FeT at different calcination temperatures (FeT3 = 300 °C, FeT4 = 400 °C, FeT5 = 500 °C).

PL spectra of TiO_2_, Fe_3_O_4,_ and 0.025FeT NCs are shown in Fig 1(a). The PL exhibits excitation-dependent properties of the synthesized material. The broad emission for all samples occurred between the range of 330–550 nm which shows the absorption edge of undoped or bare TiO_2_ and 0.025FeT (calcined at 300 °C, 400 °C and 500 °C) at 360 nm, respectively, while the absorption edge of 0.025FeT NCs was found between both wavelengths. [47,48]. In addition, photoluminescence spectra reflected the photogenerated carrier transfer and recombination behavior of the catalysts. A lower photoluminescence intensity revealed inhibited recombination of photogenerated electrons and holes, thus facilitating an enhanced utilization rate of photogenerated electrons and holes at the interface. Therefore, the charge separation efficiency was studied under an excitation wavelength of 385 nm for all catalyst samples. As shown in Fig 1(a) bare TiO_2_, 0.025FeT4 and 0.025FeT5 exhibited a higher PL intensity signal, indicating a faster recombination rate of electron-hole pairs. The PL intensity signals of 0.025FeT3 showed relatively low, indicating that the introduction of Fe_3_O_4_ into the bare TiO_2_ enhanced the separation ability of photogenerated e^−^-h^+^ pairs, making them less prone to recombination. The lower PL intensity signal could also be attributed to the controlled orientation of Fe_3_O_4_ nanocrystals on a specific crystal plane. This unique surface structure provided a larger contact area with TiO_2_ nanoparticles, which promoted the transfer of photogenerated electrons from TiO_2_ nanoparticles to Fe_3_O_4_ nanocrystals. The photogenerated electron-hole pairs had higher separation efficiency and greater photocatalytic activity. The introduction of Fe_3_O_4_ into TiO_2_ altered the photogenerated carrier transfer pathway by forming Ti-O-Fe bonds, thereby inhibiting the recombination and enhancing the catalytic activity of the catalyst [48,49].

#### 3.1.2. XRD analysis.

In the XRD plot of 0.025FeT NCs (Fig 1b) calcined at 300 °C (FeT3), 400 °C (FeT4), and 500 °C (FeT5), the main peaks observed correspond to different crystallographic planes, indicating the presence of specific crystalline phases. Temperature-dependent structural and phase shifts can be observed using XRD analysis of magnetic TiO_2_ nanocomposites (FeT3, FeT4, FeT5) calcined at 300 °C, 400 °C, and 500 °C. At 300 °C (FeT3), anatase TiO_2_ is dominant, with a large peak at ~25.3° (2θ) for the (101) plane, indicating low crystallinity and tiny crystallite sizes (17.71 nm). There are some weak Fe_3_O_4_ peaks, such as ~30.1°, ~ 35.5°, and ~57.2° for the (220), (311), and (511) planes. At 400 °C (FeT4), the crystallinity enhances, exhibiting more defined anatase peaks and a slight formation of rutile (~27.5° (2θ) for the (110) plane). The peaks of Fe_3_O_4_ become more pronounced, indicating improved crystal growth and stability. At 400 °C, the crystallite size of FeT4 reduces to 5.58 nm, suggesting particle refinement. At 500 °C, FeT5 exhibits high crystallinity, with strong anatase peaks and stable Fe_3_O_4_ reflections. There is no noticeable phase transition from anatase to rutile; the sharper peaks indicate increased crystallite sizes (7.99 nm). The findings demonstrate that anatase TiO_2_ is stable up to 500 °C, with just minor rutile formation. Fe_3_O_4_ retains its phase integrity and crystalline structure with increasing temperature [21,25]. The gradual changes in crystallite size, peak intensity, and phase composition are due to the interaction of calcination temperature, crystal growth, and phase stability.

#### 3.1.3. FTIR analysis.

The FTIR analysis (Fig 1c) reveals the chemical structure and phase transition of 0.025FeT NCs. The intensity and sharpness of the peaks associated with Fe-O and Ti-O bonds increased with increasing calcination temperature, indicating improved crystallinity. Peaks associated with hydroxyl groups and adsorbed water reduced with increasing temperature, indicating surface dehydration and better crystallization. For sample 0.025FeT3, the peaks associated with O-H and H_2_O groups are likely more pronounced, indicating more adsorbed water and hydroxyl groups. The Fe-O peaks are present, but less intense, indicating reduced crystallinity at this temperature. The strength of the Fe-O and Ti-O peaks increases with higher calcination (0.025FeT4), indicating improved crystallinity. The desorbed water causes the O-H and H_2_O peaks to decrease. At 500 °C, 0.025FeT5 exhibited strong peaks matching to Fe-O and Ti-O bonds, indicating enhanced crystallinity and phase change (anatase to rutile for TiO_2_). High temperatures cause the elimination of surface hydroxyl groups and adsorbed water, resulting in low O-H and adsorbed water peaks [25].

#### 3.1.4. Vibrating Sample Magnetometry (VSM).

The magnetic properties of calcined FeT NCs were analyzed using VSM as shown in Fig 1d. By analyzing the hysteresis loop, critical parameters such as saturation magnetization (M_s_), coercivity (H_c_), and remanent magnetization (Mr) were derived to understand the magnetic interactions within the nanocomposites and how they evolve with varying calcination temperatures. The results indicate that all FeT calcined at three different calcination temperatures 300 °C, 400 °C and 500 °C, exhibited soft magnetic behavior, close to superparamagnetism, characterized by negligible remanence and coercivity at room temperature. This soft magnetic behavior is attributed to the presence of Fe_3_O_4_ nanocrystals, which prevent magnetic ordering and allow for rapid magnetization and demagnetization. Additionally, variations in calcination temperature influence the saturation magnetization (M_s_), with the FeT5 sample exhibiting the highest M_s_ value (10.04 emu/g), followed by FeT3 (9.104 emu/g) and FeT4 (6.052 emu/g). FeT3 has a lower saturation magnetization of 9.104 emu/g, but it is still significantly higher than FeT4. The decline in magnetization at lower calcination temperatures can be linked to incomplete crystallization [21]. Despite this, the magnetic nature of FeT nanocomposites facilitates easy magnetic separation, making them highly suitable for photocatalytic applications requiring recyclability and reusability. Pure Fe_3_O_4_ exhibits superparamagnetic behavior (reported in our previous study: 61.7 M_s_, 70.1 H_c_, and 5.61 M_r_) [50]. Although calcination at higher temperatures can lower magnetization due to oxidation to maghemite (γ-Fe_2_O_3_) or hematite (α-Fe_2_O_3_). Coating Fe_3_O_4_ with a non-magnetic shell, such as TiO_2_, often reduces saturation magnetization due to dilution and magnetic shielding [30]. Calcinating FeT NCs at 300, 400, and 500 °C resulted in soft magnetic behavior with low coercivity and remanence due to Fe_3_O_4_ nanocrystals. Saturation magnetization varied with calcination temperature, with FeT5 having the highest M_s_ (10.04 emu/g), indicating enhanced crystallinity, and FeT4 displaying the lowest, possibly due to incomplete crystallization. The magnetic characteristics allow for facile separation and reuse, however, the presence of a TiO_2_ shell and thermal oxidation may reduce magnetism compared to pure Fe_3_O_4_ NP. The samples displayed soft magnetic behavior, close to superparamagnetism, with near-zero coercivity and remanence. The data aligns with the magnetic properties of already reported FeT NCs [51].

#### 3.1.5. Bandgap Estimation Analysis (DRS).

**Fig 2:**
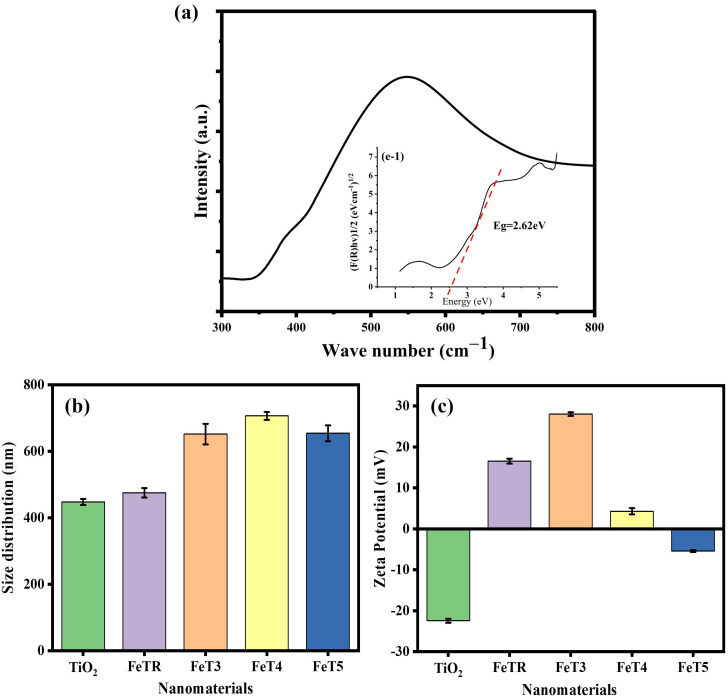
(a) Tauc plot of 0.025FeT-3 photocatalyst (b) Hydrodynamic Size and Zeta Potential of FeT at different calcination temperatures (FeT3 = 300 °C, FeT4== 400 °C, FeT5 = 500 °C).

Modifying the lattice structure of titanium dioxide (TiO_2_) by incorporating impurities enhances its ability to function under visible light. In this study, diffuse reflectance spectroscopy (DRS) was utilized to analyze this shift and estimate the bandgap. The absorption spectra of 0.025FeT3 (FeT3) presented in Fig 2a demonstrate a notable transition towards the visible spectrum. This increased absorption of visible light is attributed to the presence of Fe_3_O_4_ and the formation of impurity levels within the TiO_2_ lattice, which facilitate electron excitation at lower energies. The optical bandgap of the composite, determined using the Tauc plot (inset, which is a graph of (F(R)hv)¹/² versus hv), was found to be 2.62 eV, confirming the significant redshift from the UV range into the visible spectrum (absorption onset, around 473 nm). Pure TiO_2_ (anatase) typically exhibits a sharp absorption edge at approximately 390 nm, corresponding to a bandgap of 3.20 eV [52]. The reduction in bandgap by the incorporation of Fe_3_O_4_ as an impurity to modify the lattice structure of titanium dioxide is assigned to function as an electron acceptor, facilitating charge carrier separation and reducing recombination losses. Previous studies report bandgaps for Fe_3_O_4_/TiO_2_ nanocomposites ranging between 2.65–2.89 eV, depending on Fe_3_O_4_ concentration, annealing temperature, synthesis pH and synthesis method. [53,54]. To strengthen this statement, the BET results have been previously reported in our publication [55] revealing that Fe_3_O_4_ exhibits a surface area of 17.507 m^2^/g along with larger pore sizes, measuring 1.8543 nm.

#### 3.1.6. Hydrodynamic Size and Zeta Potential.

At neutral pH, TiO_2_ in water exhibits a hydrodynamic size of 447.5 ± 9.15 nm and a zeta potential of −22.4 ± 0.5 mV. This indicates good stability and resistance to agglomeration due to electrostatic repulsion. Absolute values of zeta potential greater than or equal to 30 mV are typically seen as strongly stable; however, the zeta potential measured here suggests moderate stability, which is adequate to help prevent agglomeration through electrostatic repulsion and possible steric effects from the surrounding solvent [56]. When Fe_3_O_4_ is doped onto the titania, the hydrodynamic radius increases to 475 ± 14.2 nm, and the zeta potential shifts to a positive value of 16.5 ± 0.6 mV, indicating a slight decrease in dispersion stability. Following calcination at 300 °C, the zeta potential of the FeT rises to +28.03 ± 0.5 mV, while the hydrodynamic radius increases to 651.5 ± 30.9 nm, suggesting particle agglomeration during calcination, yet moderately improved electrostatic stabilization. As the calcination temperature increases further, the hydrodynamic radius expands to 706.3 ± 11.8 nm, and the zeta potential decreases to +4.31 ± 0.4 mV, indicating enhanced agglomeration. At even higher temperatures, the hydrodynamic radius slightly decreases to 654 ± 23.7 nm, and the zeta potential changes to −5.41 ± 0.27 mV, signifying instability in the nanoparticle dispersion. These alterations reflect increased crystal growth and agglomeration with calcination, as supported by the observed trends in hydrodynamic size and surface charge shown in Fig 2b and Fig 1(c).

#### 3.1.7. XPS analysis.

**Fig 3:**
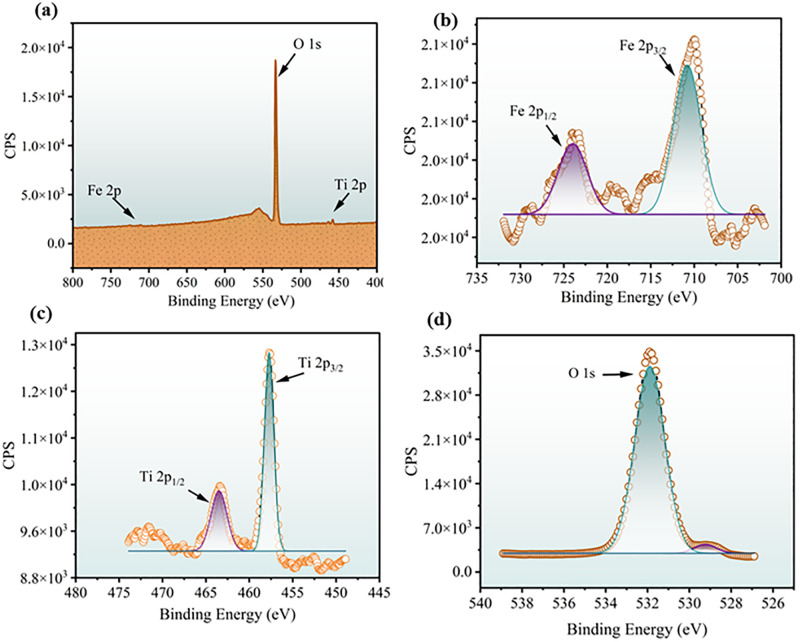
XPS spectra of 0.025FeT3 (FeT3) photocatalysts: (a) survey,(b) Fe 2p, (c) Ti 2p, and d) O 1s.

XPS was used to investigate the surface chemical composition and oxidation states of the 0.025FeT3 (FeT3) photocatalysts (Fig 3). The survey spectrum (Fig 3a) confirmed the presence of Fe, Ti, and O elements without any detectable impurities, demonstrating the successful synthesis of the binary oxide composite. The Fe 2p high-resolution spectrum (Fig 3b) exhibited two major peaks at ~710.8 eV (Fe 2p_3/2_) and ~724.5 eV (Fe 2p_1/2_), corresponding to Fe³^+^ species in Fe_3_O_4_/Fe_2_O_3_. The additional shake-up satellite features observed in the Fe 2p region are typical signatures of Fe^3+^, arising from charge-transfer excitations, and indicate the coexistence of redox-active Fe^2+^/Fe^3+^ centers [57]. Such Fe^3+^ states are particularly important in photocatalysis because they can trap photogenerated electrons, suppress electron–hole recombination, and promote the formation of reactive oxygen species (ROS), thereby enhancing dye degradation [58].

The Ti 2p spectrum (Fig 3c) showed two well-resolved peaks at ~458.6 eV (Ti 2p_3/2_) and ~464.3 eV (Ti 2p_1/2_), with a spin-orbit splitting of ~5.7 eV, characteristic of Ti^4+^ in TiO_2_ [58]. This confirms that titanium is fully oxidized, thereby providing strong oxidative potential for photocatalytic activity. The O 1s XPS spectrum (Fig 3d) shows a peak at 530.1 eV, indicating lattice oxygen (O^2–^) with Fe–O and Ti–O bonds. In addition, the shoulder at 531.8 eV is attributed to surface hydroxyl groups or adsorbed oxygen species. These surface hydroxyls are important in photocatalysis because they can interact with photogenerated holes to produce hydroxyl radicals (•OH), which are key active species for degrading organic dyes [59].

Furthermore, a notable feature at approximately 560 eV in the survey spectrum was identified as the O KLL Auger peak, arising from non-radiative relaxation processes associated with oxygen. The existence of this specific signal is typical of a metal oxide matrix and aligns with the substantial Fe–O and Ti–O coordination present within the nanocomposite. This oxygen-rich chemical environment, intrinsic to the oxide structure, is beneficial for photocatalysis, as it offers a high concentration of active surface sites that boost reactivity and support the ongoing generation of reactive oxygen species (ROS) [60].

#### 3.1.8. BET surface area analysis.

**Fig 4:**
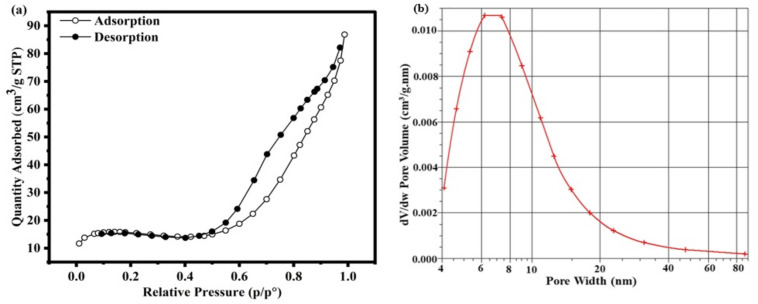
Surface area analysis of 0.025FeT3 (FeT3) photocatalysts:(a) Isotherm Linear Plotand (b) BJH Adsorption dV/dw Pore Volume.

The nitrogen adsorption–desorption isotherm of the synthesized 0.025FeT3 (FeT3) photocatalysts (Fig 4a) displays a distinctive type IV profile accompanied by an H_3_ hysteresis loop, indicating the development of mesoporous structures [48] characterized by slit-shaped pores. At low relative pressures (p/p° < 0.3), there is a gradual N_2_ uptake, which points to monolayer adsorption. In contrast, a significant uptick in adsorbed volume between p/p° ≈ 0.4–0.8 illustrates capillary condensation occurring within the mesopores. The BET analysis determined a specific surface area of 57.39 m^2^ g ^‒1^, closely matching the single-point value of 56.12 m^2^/g ^‒1^, while the Langmuir model provided a marginally elevated value of 68.13 m^2^/g ^‒1^. The t-plot analysis revealed a micropore area of 63.86 m^2^/g ^‒1^ with minimal external surface contribution, indicating that the majority of adsorption sites are located within the pores. The total pore volume at p/p° = 0.987 was found to be 0.134 cm^3^ g ^‒1^, with a micropore volume of 0.0267 cm^3^ g ^‒1^, emphasizing the predominance of mesopores in the textural structure. The average pore diameter, as calculated by the BET method, was 9.36 nm (adsorption) and 9.19 nm (desorption), corroborated by BJH analyses, which indicated average widths of 11.33 nm (adsorption) and 7.83 nm (desorption).

The BJH pore size distribution, derived from the adsorption branch, further confirmed the mesoporous nature of FeT3. The dV/dw plot (Fig 4b) displayed a pronounced peak at ~6–8 nm, with a maximum pore volume increment of approximately 0.011 cm^3^ g ^‒1^ nm ^‒1^, indicating that the highest concentration of pores is centered in this range. The pore volume contribution declined gradually beyond 10 nm, with negligible macropore presence above 80 nm. This distribution is consistent with the BJH average pore width of 11.33 nm (adsorption) and 7.83 nm (desorption) and the BET-derived pore diameters of 9.36 nm (adsorption) and 9.19 nm (desorption). Together, these findings demonstrate that the FeT3 has a moderately high surface area and a well-developed mesoporous framework, with pores mainly concentrated in the 6–8 nm region and minimal microporosity [61]. Such a uniform mesostructure provides ample accessibility to reactant molecules and facilitates the efficient dispersion of active sites, thereby enhancing the material’s potential for photocatalytic degradation of dyes and other organic contaminants [18]. These observations reinforce the successful modification of TiO_2_’s electronic structure, enabling it to absorb light in the visible range. This enhancement makes TiO_2_ a promising candidate for solar-driven photocatalysis. Additionally, surface area and pore size are crucial factors, as they indicate that the nanoparticles (NPs) and related nanocomposites (NCs) provide more accessible surface areas for adsorbing gases or liquids due to their increased pore sizes [62]. As a result, the 0.025FeT3 nanocomposite accomplished a 84.51% removal of RY145 in 60 minutes, achieving complete degradation within 70 minutes under visible light, illustrating a strong link between the textural properties of the surface and photocatalytic efficiency.

#### 3.1.9. Surface morphology analysis.

**Fig 5:**
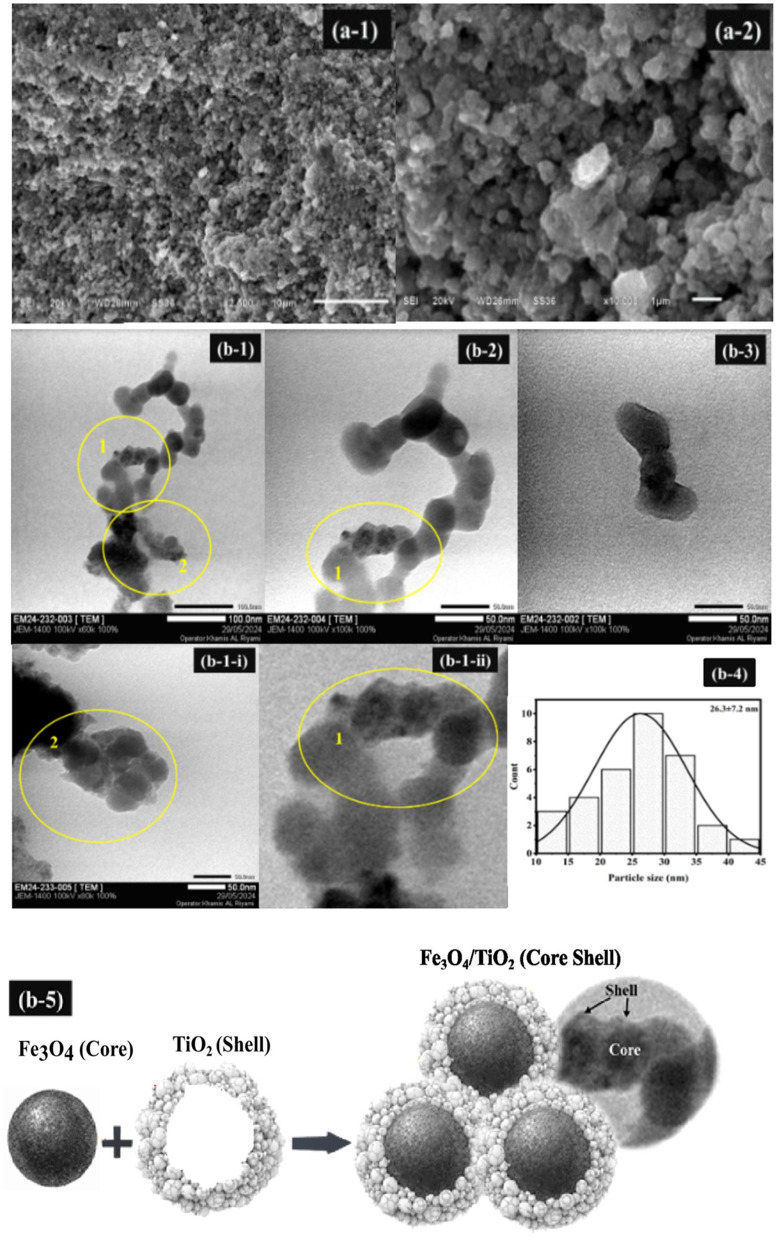
Surface morphological analysis of 0.025FeT3: (a-1 to a-2) SEM and, (b-1, b-2, b-3) TEM images showing aggregated and chain-like assemblies of quasi-spherical nanoparticles; (b-1-i and b-1-ii) TEM images highlighting contrast variation between darker Fe_3_O_4_ domains and lighter TiO_2_ surface regions, indicating a core-shell-like heterostructure and Ti-O-Fe interfacial junction; (b-4) Particle size distribution histogram (b-5) A core-shell structural model.

Surface and morphological features of 0.025FeT3 were studied using SEM-EDS (Fig 5a, S12) and TEM analyses (Fig 5b). In TEM micrographs, particle aggregation was observed as multiple nanocrystals joined together to form a larger particle. The SEM image of the 0.025FeT3 nanocomposite indicates that TiO_2_ nanoparticles were attached to larger micrometer-sized Fe_3_O_4_ nanoparticles, forming a coating over the surface of the Fe_3_O_4_ particles. In general, such aggregates can provide insights into the structure and composition of the particles. In the Energy Dispersive X-ray (EDS) spectrum, the carbon (C) signal is most likely to originate directly from the carbon-coated copper grid, supporting carbon film, or tape that was used to prepare the SEM sample. Such contaminants are not indicative of any contamination in the yielded FeT NCs and are typical sources of background carbon signals. TEM micrographs (Fig 5b) show that the 0.025FeT3 is made up of quasi-spherical nanoparticles with an average size of ~20−30 nm (mean: 26.3 ± 7.2 nm, Fig 5b4), forming aggregated and chain-like assemblies. Fig 5b-1 and Fig 5. b-2 reveal dense agglomerates. In contrast, magnified images of Fig 5. b-1 (as Fig 5b-1-i and Fig 5. b-1-ii) reveal that these clusters are made up of loosely packed nanograins with distinct borders, indicating incomplete coalescence. Fig 5b-1-i and Fig 5. b-1-ii show contrast differences inside individual particles, indicating darker core areas and lighter outside layers due to Fe_3_O_4_ and TiO_2_, respectively. The contrast difference suggests the existence of a core-shell heterostructure, in which individual crystallites of TiO_2_ are distributed as a surface coating over Fe_3_O_4_ instead of a completely homogeneous shell. This leads to a porous and diverse surface layer. This configuration is classed as an aggregated heterostructure or nanocomposite because of the intimate interfacial contact between the Fe_3_O_4_ and TiO_2_ phases. Several reasons contribute to the observed aggregation, including Fe_3_O_4_’s magnetic characteristics, which favor particle clustering, Van der Waals interactions between nanoparticles, and the unique synthesis conditions.

The interfacial region can be considered a Ti-O-Fe junction, as previous analyses in sections 3.1.1 and 3.1.7 (PL and XPS analysis) have indicated significant Fe-O and Ti-O coordination. This shows a strong interfacial interaction between the two phases. Furthermore, magnetic interactions between Fe_3_O_4_ domains are likely to contribute to the observed chain-like aggregation (Figs 5b1 to 5b3), showing the presence of these domains in the composite. The TEM analysis shows the creation of a core-shell-like Fe_3_O_4_/TiO_2_ architecture, but with a heterogeneous structure rather than an ideal, well-defined core-shell system. Large particle aggregates in TEM images of 0.025FeT3 arise from factors such as the synthesis process (high temperatures or rapid precipitation), the magnetic properties of Fe_3_O_4_ that encourage clustering, and inadequate surface functionalization leading to agglomeration. Sample preparation issues like poor dispersion or rapid drying also contribute. Additionally, the choice of solvent and stabilizing agents impacts particle dispersion, with larger particles being more susceptible to aggregation. Controlling these factors can enhance the dispersion of 0.025FeT3 [62].

TEM (Fig 5) was used further to characterize the morphological characteristics of the 0.025FeT3 nanocomposites. The results showed the formation of loosely aggregated, nearly spherical nanoparticles with an average size of approximately 10–20 nm. The TEM image’s porous network indicates the presence of mesoporosity and interparticle voids, both of which enhance photocatalytic activity by providing more accessible active sites. Furthermore, the XRD patterns showed broad diffraction peaks (Fig 1b), implying a nanocrystalline structure and supporting the presence of small particle sizes and high surface area features [62]. These TEM and XRD results qualitatively suggest that the produced 0.025FeT3 nanocomposites probably have a high specific surface area and a mesoporous character, even though BET analysis was unavailable in the current investigation. These structural features are believed to aid in the efficient adsorption of dye molecules as well as the separation of photogenerated charge carriers, hence contributing to the reported outstanding photocatalytic activity under visible light exposure.

#### 3.1.10. Thermogravimetric analysis (TGA).

Thermogravimetric analysis (TGA) of the uncalcined 0.025FeT NCs was conducted using a PerkinElmer Pyris instrument to assess its thermal stability and decomposition behavior with rising temperature. A 5.300 mg sample was heated from 35°C to 800°C at a constant rate of 10°C/min under the air atmosphere. The TGA curve (S6 Fig.) displayed a total weight loss of about 13.983%, occurring in three distinct stages. The initial minor loss below 120°C corresponds to the disruption of physically adsorbed moisture and residual volatile solvents. The gradual mass decrease observed between 120°C and 500°C is mainly attributed to the removal of surface hydroxyl groups, decomposition of residual organic species, and oxidation of remaining precursors by-products. Beyond 500°C, a slow and continuous weight decline up to 800°C suggests dehydroxylation, condensation of surface groups, and lattice rearrangement processes. The absence of any abrupt or large-scale decomposition steps indicates that the nanocomposite possesses good thermal stability. These results support that calcination within 450–550°C is optimal to eliminate organic residues and hydroxyl groups, promoting enhanced crystallinity and phase purity while maintaining the anatase structure and Fe_3_O_4_ stability essential for efficient photocatalytic activity.

### 3.2. Discoloration studies – screening and optimization studies

**Fig 6:**
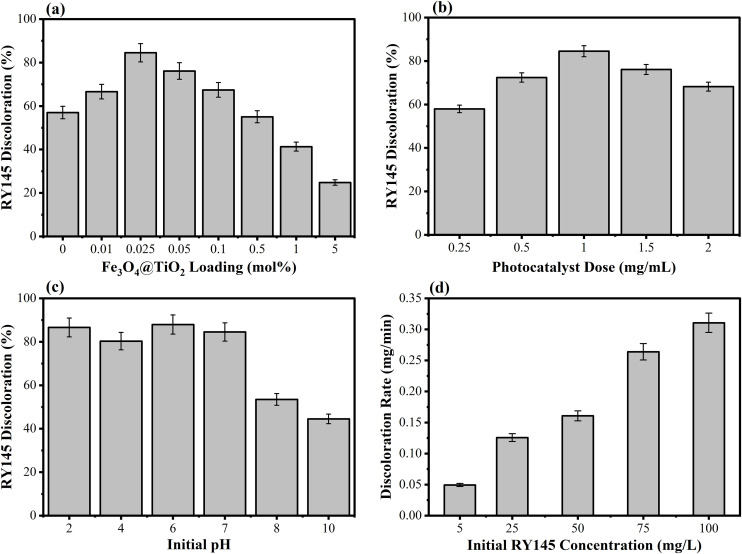
Photocatalytic degradation studies of 0.025FeT3: Effect of (a) Fe_3_O_4_ contents (mol%); (b) photocatalyst dose; (c) initial pH; (d) rate of discoloration against initial RY145 concentration. Reaction Conditions: Reaction temperature 22 ± 3 °C, photocatalyst dose 1 mg mL^-1^, pH 6.8–7, illumination 500 W halogen lamp, irradiation time 60 min.

Experiments were conducted with different contents of Fe_3_O_4_ and TiO_2_ (i.e., 0.01, 0.015, 0.025, 0.035, 0.05, 0.1, 0.5, 1, and 5 mol% at three difficult calcination temperatures, 300 °C, 400 °C, and 500 °C. Fig 6 shows 0.025FeT3 as the optimum loading for 84.51% % RY145 discoloration during screening studies. In a photocatalytic reaction, e^-^/h^+^ pair generation is promoted in 0.025FeT NCs. For the photocatalytic reaction, the suspension was subsequently exposed to direct visible light irradiation. Superoxide ions (O_2_-) are created when the excited electrons interact with the dissolved molecular oxygen. Hydroxide radicals (OH) are created when the photogenerated holes (*h*^+^) in the valence band combine with the water molecules and hydroxide (OH^-^) ions. The RY145 dye in the water can be discolored by the extremely reactive radicals O_2_^-^ and •OH [12,63,64]. For the photocatalytic degradation of RY145, the data in Fig 6a highlight the influence of Fe_3_O_4_ loading (mol%) on TiO_2_. Low loadings (0–0.01 mol%) result in little dye elimination (~16.6%), suggesting that the Fe_3_O_4_ level is insufficient to considerably boost photocatalytic activity. The optimum performance was reported at 0.025 mol% Fe_3_O_4_, with the removal of dye reaching 84.51%, with the maximum degradation in 60 minutes, which further reached 100% in 70 minutes of visible light irradiation, demonstrating a strong synergistic relationship between Fe_3_O_4_ and TiO_2_. However, beyond this optimum, a surged Fe_3_O_4_ content leads to a considerable drop in degradation efficiency, notably at 1–5 mol%, likely due to excessive Fe_3_O_4_ blocking TiO_2_’s active sites and light absorption and promoting charge carrier recombination. Controlling Fe_3_O_4_ content precisely is crucial for improving photocatalytic activity, according to these findings. Although the degradation efficiency of RY145 using 0.025FeT3 is 84.51% % is lower than other systems reporting ~99% removal [23], it is important to consider that the experiment was conducted without sacrificial agents, additional oxidants, or pH adjustments, under visible light and neutral pH conditions, which reflect more realistic environmental applications. Notably, degradation efficiency reaches 100% after 70 minutes, while the results presented are standardized for consistency at 60-minute intervals of irradiation.

The selectivity of the FeT photocatalyst was evaluated by conducting degradation tests with different dyes, including RY145, Methyl Orange (MO), and Reactive Black 5 (RB5). The catalyst exhibited high removal efficiencies of 84.51%, 96.8%, and 91.6% for RY145, MO, and RB5, respectively, demonstrating its broad-spectrum photocatalytic activity and robustness toward dyes of varying molecular structures and charges. These results (S7 Fig.) confirm the catalyst’s strong selectivity and efficiency in treating mixed dye systems, indicative of its potential for practical wastewater applications.

#### 3.2.1. Effect of nanocomposite dose.

The optimization of photocatalyst dose (0.025FeT3) is a significant issue as it prevents the use of excessive nanomaterials and ensures effective light absorption. Fig 6b shows the effect of a different 0.025FeT3 dose on the RY145 discoloration. In control experiments, only light and without the addition of photocatalyst, very little decolorization (1.2%) was observed, but it increased from 57% to 84.51% when the dose of 0.025FeT3 increased from 0.25 to 1.0 g/L^,^ respectively. Moreover, upon further increasing the photocatalyst dose from 1.0 to 2.0 g/L^,^ the discoloration efficiency decreased from 84.51% to 68.19%, respectively. Such findings are usually shown by the most photocatalytic reactions. Basically, the increase in dose gives more active sites on the photocatalyst (0.025FeT3) surface and hence increases the RY145 discoloration; however, further increase in dose increases the turbidity of the reaction medium and results in the scattering of the light irradiation, which in turn decreases the discoloration efficiency. Some research findings also showed that agglomeration of the photocatalysts at a higher amount of dose is responsible for low discoloration efficiency due to the loss of surface area and the low penetration of sunlight [65,66].

#### 3.2.2. Effect of reaction pH.

The effect of variable pH on the RY145 discoloration efficiency of 0.025FeT3 is shown in Fig 6c. While the discoloration process is influenced by pH, the photocatalyst’s interaction with the target organic pollutants can also cause interference with the photocatalytic process in the wastewater treatment system. Decolorization efficiency does not change significantly between pH 2 to pH 6. However, lower RY145 discoloration (44.51%) was observed at pH 10. Acidic pH is more favorable for the discoloration of RY145 dye using 0.025Fe_3_O_4_-TiO_2_-300 (0.025FeT3) compared to basic pH. At lower pH the 0.025FeT3 surface is positively charged, RY145 is an anionic dye possessing a negative charge due to dissociation of sulfonic groups, resulting in repulsion between the RY145 dye molecules and 0.025FeT3, which in turn causes negligible adsorption at higher pH and strong attraction at lower pH, leading to high discoloration of the RY145 dye [63]. Similar results were also reported in previous studies for anionic dyes and the higher photocatalytic discoloration of RY145 at acidic pH [67–69].

#### 3.2.3. Photocatalytic adsorption and kinetic studies.

**Fig 7:**
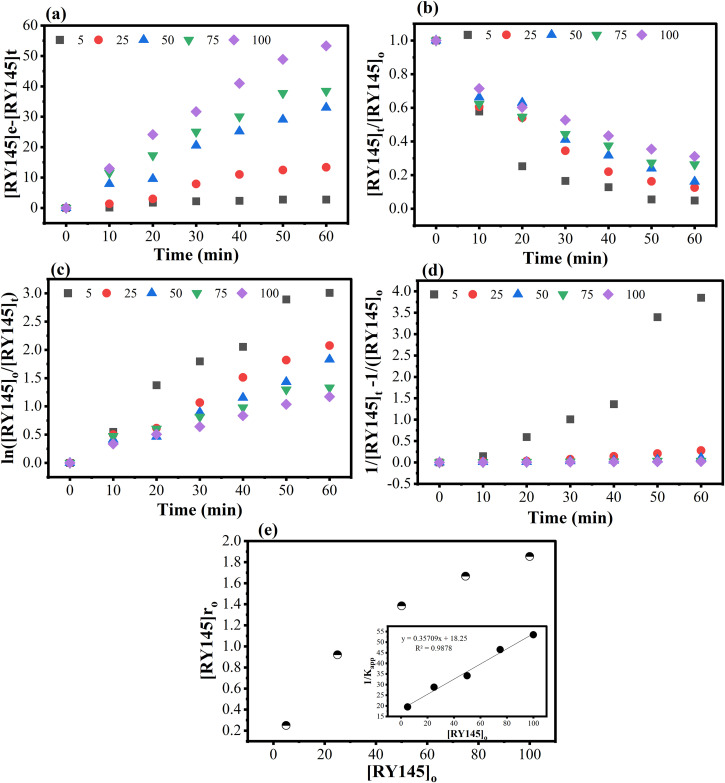
(a) Zero order, (b) FO, (c) PFO, (d) SO and (e) Effect of RY145 concentration on the initial rate of RY145 decolorization, inset: Plot of reciprocal of apparent rate (K_app_) of discoloration against the initial concentration of RY145.

The impact of the initial concentration of RY145 on the discoloration efficiency of the 0.025FeT3 photocatalyst was examined by altering the concentrations (5, 25, 50, 75, and 100 mg L^-1^). The discoloration efficiency was found to decline as the pollutant load increased, ranging from 95.06% at 5 mg L^-1^ to 68.94% at 100 mg L^-1^ as shown in Fig 6d. Kinetic analysis of the discoloration data (as in Fig 7a Zero order, (b) FO, (c) PFO, and (c) SO) indicated that the reaction was best described by the pseudo-first order (PFO) model, suggesting that the photocatalytic degradation of RY145 adheres to PFO kinetics. At lower dye concentrations, a higher discoloration efficiency is noted due to the increased availability of active sites for both adsorption and photocatalytic activity [70]. Conversely, as the concentration of RY145 rises, these active sites become occupied, leading to a decrease in photocatalytic effectiveness. Furthermore, elevated dye concentrations result in photon blockage and a reduced generation of •OH and •O_2_ radicals, attributed to the adsorption of dye on the surface of the photocatalyst [71,72].

Adsorption equilibrium data for RY145 on 0.025FeT-3 NCs were investigated using Langmuir and the Freundlich isotherm models (S7 Fig.) to understand the adsorption mechanism. The Langmuir model yielded a high correlation coefficient (R² = 0.9968), indicating that dye adsorption primarily occurs in a monolayer pattern over a homogenous surface. The maximum adsorption capacity (*K*__𝑎𝑑_𝑠_) was 38.02 mg g^‒1^, and the Langmuir constant (*K*__𝑎𝑑_𝑠_) was 0.00819 L mg^‒1^, indicating a moderate adsorption affinity between the dye molecules and the photocatalyst surface. The Freundlich model had a bit lower correlation coefficient (R² = 0.9115), with 𝐾_𝐹_ = 1.85 and 𝑛 = 1.95, suggesting favorable but heterogeneous multilayer adsorption. The superior fit of the Langmuir model shows that RY145 dye adsorption on 0.025FeT-3 happens mostly via monolayer adsorption onto energetically uniform sites, which is consistent with the composite’s mesoporous structure as demonstrated by BET analysis.

The L-H kinetic model was utilized to analyze the kinetics of the photocatalytic reaction. The theoretical assessment of photo discoloration at varying RY145 concentrations can be derived from the L-H model using Equation (3). The inset of Fig 7e validates the L-H relationship by plotting 1/K_app_ against [RY145]₀. The K_c_ value was determined to be 2.80 mg L min^-1^ while the K_LH_ value was 2.42 L mg^-1^. The elevated K_c_ value suggests that the photocatalytic process is more influential than the adsorption process [72]. Results from the L-H model indicate that the heterogeneous photocatalytic process encompasses both adsorption and photocatalysis, which occur concurrently [73]. This simultaneous process is further illustrated in Fig 7e, where the rate of photo-discoloration increased with the initial concentration of RY145 (up to 100 mg L^-1^) but started to decline as the active sites became saturated.

### 3.3. Photocatalytic mineralization and mechanism investigation

**Fig 8:**
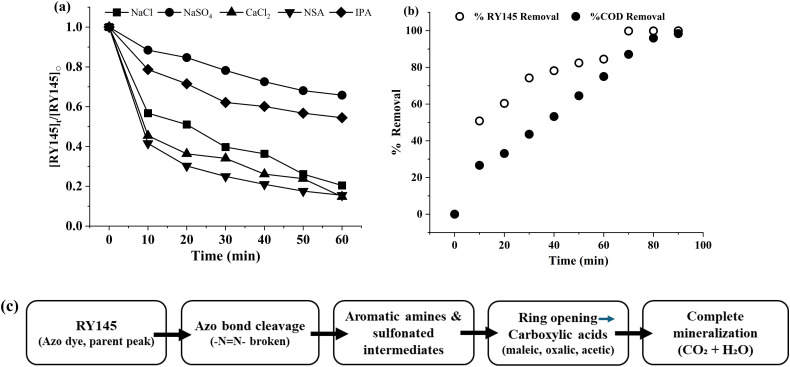
(a). Photocatalytic mechanism investigation using scavenging species (b) Percent discoloration vs mineralization (COD analysis) (c) Sketch 1: RY145 photocatalytic degradation pathway.

The active species trapping experiments were carried out to identify and assess the role of various reactive species in the photocatalytic process. Scavengers or traps are used to suppress certain species involved in photocatalysis, such as hydroxyl radicals (•OH), superoxide anions (O_2_•^‒^), and holes (h^+^), to determine which species are most important for the discoloration process. Overall, discoloration trends varied amongst traps. The maximum RY145% removal presented in Fig. 8(a) was obtained for the reaction with CaCl_2_ (85.24% removal), without any scavenging agent (NSA, 84.51%) and NaCl (79.57%). There was minimal deterioration with Na_2_SO_4_ (34.19%) and IPA (45.54%).

In photocatalytic processes, •OH can be scavenged by NaCl and IPA. The relatively significant dye removal percentage (79.57%) observed with NaCl suggests that •OH radicals play a crucial role in the photocatalytic process. Although NaCl scavenges •OH, the high removal rate suggests that other active species, such as holes (h^+^) or superoxide anions (O_2_•^‒^), may also play a significant role in the discoloration process. IPA has a lower removal efficiency (45.54%) compared to NaCl and CaCl_2_, indicating that while it reduces the activity of hydroxyl radicals, the degradation may not be entirely due to •OH. Other species may also be important in the photocatalytic degradation, such as holes (h^+^) or superoxide anions (O_2_•^‒^).

Sulfate radicals (SO_4_•^‒^), which are implicated in certain photocatalytic processes, are the main target of Na_2_SO_4_. When compared to NaCl, the relatively low removal (34.19%) with Na_2_SO_4_ suggests that SO_4_• ^‒^ radicals may not be as important for dye degradation in this system, or that their contribution is less significant than that of •OH or h^+^ radicals. CaCl_2_, which scavenges hydroxyl radicals (•OH) and releases Ca²^+^ ions that interact with the catalyst surface, shows a high removal rate, indicating that the degradation process is predominantly driven by •OH radicals. This emphasizes the role of •OH in the photocatalytic process. The experiment with no scavenging agents (NSA, 84.51%) is used as a baseline when none of the scavengers are added. The high removal efficiency with NaCl and CaCl_2_ suggests that hydroxyl radicals (•OH) are the most significant or the main active species in the photocatalytic process, according to the findings. The moderate results obtained from Na_2_SO_4_ (sulfate radical scavenger) and IPA (•OH scavenger) imply that superoxide anions and h^+^ contribute to the discoloration, though their role is secondary compared to •OH. The overall photocatalytic mechanism is most likely driven by a combination of hydroxyl radicals, holes, and potentially superoxide anions, with hydroxyl radicals playing the primary role. The PL data (Fig. 1a) indicate that TiO_2_ doped with Fe³^+^ exhibits reduced photoluminescence intensity compared to undoped TiO_2_, which suggests lower electron-hole recombination. Moreover, scavenger experiments indicate that hydroxyl radicals (•OH) are the main reactive species involved in dye degradation. Collectively, these findings support the suggested mechanism whereby Fe³^+^ captures electrons, leading to increased formation of reactive oxygen species (ROS) and enhanced photocatalytic activity.The impact of substrate type (S8 Fig.) on photocatalytic efficiency was assessed using three representative dyes: RY145, Reactive Black 5 (RB5), and Methyl Orange (MO). The 0.025FeT3 nanocomposite demonstrated varying degradation performance depending on the substrate, with MO achieving the highest removal efficiency, followed by RB5 and RY145. The superior degradation of MO can be attributed to its simpler molecular structure and greater vulnerability to oxidative attack by reactive species, while the more intricate azo structures of RY145 and RB5 result in lower degradation rates. These findings illustrate how the structure of dyes affects photocatalytic efficiency and affirm the robust oxidative capability of the 0.025FeT3 system.

The comparison of dye discoloration and COD removal reveals the difference between visible decolorization and real mineralization. Although the FeT3 achieved over 100% discoloration of RY145 within 70 minutes, COD elimination was only ~90%, indicating the presence of intermediate degradation intermediates. Fig. 8(b) reveals that decoloration is fast and almost complete (~100% in 70 minutes), but COD elimination follows behind, reaching slightly below ~90%. This demonstrates that photocatalytic activity breaks down dye molecules, but certain organic intermediates remain, necessitating longer time or improved conditions for complete mineralization. This emphasizes the significance of integrating both approaches for precise photocatalytic assessment and environmental significance.

The HPLC chromatogram (S9 Fig.) of RY145 after photocatalytic degradation with 0.025FeT3 under visible light irradiation exhibited several peaks corresponding to intermediate degradation products. The parent azo dye peak was absent, indicating that the azo bond was successfully cleaved. The early retention peaks at 6.05 and 8.20 min indicate the synthesis of short-chain carboxylic acids and polar intermediates, which is consistent with azo bond scission. Peaks at 24.71 and 28.24 min are attributable to aromatic amines and hydroxylated aromatic derivatives, which are common stages in azo dye degradation pathways. The subsequent peaks at 32.54 and 36.08 minutes correspond to dicarboxylic acids and partly oxidized aromatics, showing a gradual oxidative change. Later peaks (48–52 min) are trace-resistant species or oligomeric fragments that gradually decline with extended irradiation. These data support the suggested degradation mechanism, which involves azo bond breaking, aromatic amine production, ring-opening to carboxylic acids, and mineralization to CO_2_, H_2_O, and inorganic ions. The existence of several intermediates indicates progressive photocatalytic oxidation enhanced by hydroxyl radicals formed on the 0.025FeT3 surface under visible light (photodegradation pathway is presented as a sketch Fig. 8c).

**Fig 9:**
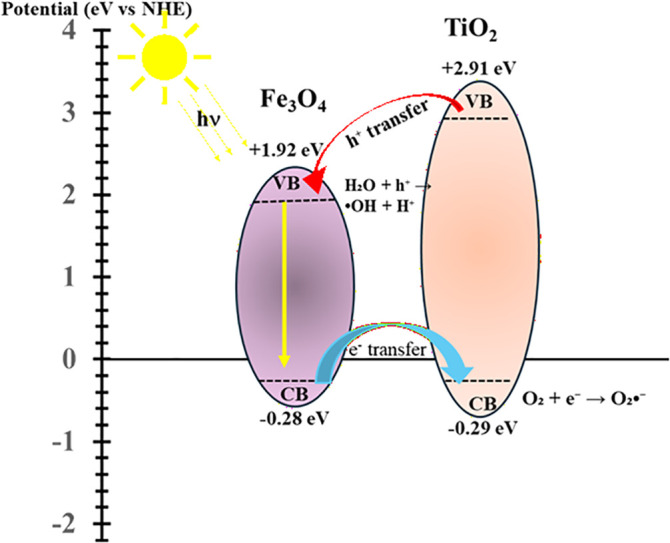
Band potential of FeT: A mechanistic diagram showing the charge-transfer path under visible light.


The band gap energies of Fe_3_O_4_ and TiO_2_ were estimated using UV–Visible Diffuse Reflectance Spectroscopy (UV–Vis DRS) followed by Tauc plot analysis. To determine the band edge potential (Fig. 9), Mulliken electronegativity theory was applied using the following equations:

$$\begin{array}{c} E_{CB}=\chi -E^{e}-\text{0}.\text{5}E_{g} \end{array}$$ (8)

$$\begin{array}{c} E_{VB}=E_{CB}+E_{g} \end{array}$$ (9)

where $E_{CB}$ and $E_{VB}$ represent the conduction and valence band potentials, respectively, χ is the absolute electronegativity of the semiconductor, $E_{e}$ is the energy of free electrons on the hydrogen scale (4.5 eV) [74]., and $E_{g}$ is the band gap energy. Using literature values of electronegativity (χ = 5.32 eV for Fe₃O_4_ and χ = 5.81 eV for TiO_2_), the calculated band edge positions are −0.28 eV (CB) and +1.92 eV (VB) for Fe_3_O_4_, and −0.29 eV (CB) and +2.91 eV (VB) for TiO_2_ vs NHE.

These results indicate the formation of a Type-II heterojunction between Fe_3_O_4_ and TiO_2_. Under visible light irradiation, Fe_3_O_4_ is preferentially excited due to its narrower band gap, generating electron–hole pairs. The photogenerated electrons migrate from the CB of Fe_3_O_4_ to the CB of TiO_2_, while holes transfer from the VB of TiO_2_ to the VB of Fe_3_O_4_. Although the CB potentials of both semiconductors are closely aligned, the electron transfer is facilitated by interfacial charge redistribution and band bending at the heterojunction interface. This spatial separation of charge carriers suppresses recombination and enhances photocatalytic efficiency. The accumulated electrons in TiO_2_ reduce dissolved oxygen to superoxide radicals (O_2_ + e^‒^ → O_2_•^‒^), while holes in Fe_3_O_4_ oxidize water or hydroxide ions to generate hydroxyl radicals (H_2_O/OH^‒^ + h^+^ → •OH), which are responsible for the degradation of dye molecules.

Scavenger tests provide only indirect evidence for the involvement of •OH or h^+^ radicals; therefore, our conclusions are presented as tentative. Future studies employing direct techniques, such as Electron Paramagnetic Resonance (EPR/ESR), fluorescence spectroscopy, or chemical reactive probes, are recommended to verify the roles of reactive species. The photocatalytic mechanism was interpreted under room-temperature (25 ± 2 °C) conditions, acknowledging that temperature-dependent kinetic studies are required in future work to accurately distinguish surface reaction and diffusion-controlled processes.

The FeT3 nanocomposite outperforms standard binary oxide systems in photocatalysis due to its interfacial structure’s synergistic effects. FeT3 composites have limited interfacial contact, which hinders effective charge transfer and causes photogenerated electron-hole pairs to recombine quickly. The mesoporous architecture established in this study allows photogenerated electrons to flow more effectively from TiO_2_’s conduction band to the Fe_3_O_4_ phase, resulting in intimate interfacial coupling between the two materials. This interfacial charge transfer reduces recombination losses and increases the formation of reactive oxygen species, specifically hydroxyl (•OH) and superoxide (O_2_•^‒^) radicals, which are principally responsible for dye degradation. The presence of Fe_3_O_4_ adds to band structure modification and extended light absorption, allowing the composite to use a wider range of the visible spectrum. The transport of RY145 molecules and reactive species is further facilitated by the mesoporous structure, increasing the overall catalytic efficiency. Compared to previously reported Fe_3_O_4_TiO_2_ systems, which often require UV irradiation [24,48], noble metal doping, or complex synthesis routes, the current nanocomposite demonstrates a more efficient and sustainable photocatalytic pathway under visible-light conditions, highlighting the importance of interfacial and structural engineering in optimizing photocatalytic systems.

### 3.4. Recycling of the photocatalysts

**Fig 10:**
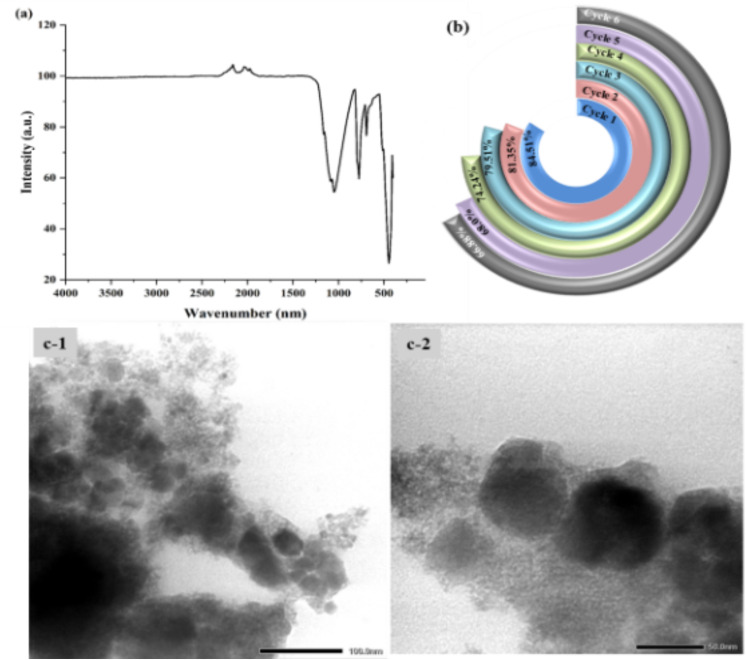
Recycling performance and post-reaction structural characterization of the 0.025FeT3 nanocomposite after the sixth cycle: (a) FTIR spectra, (b) Recycling performance for photocatalytic degradation of RY145 over consecutive cycles, (c-1, c-2) TEM images of the FeT at different magnifications.

The recycling efficiency of the 0.025FeT3 as the optimized NC for discoloration of RY145 was assessed, as described in section 2.4. The recycling performance is presented in Fig 10b as multi-layered circular doughnut chart with a gradual decrease in the photocatalyst’s effectiveness in removing RY145 dye over six cycles: 84.51% in the first run, 81.35% in the second (C2), 79.51% in the third (C3), 74.24% in the fourth (C4), 68.00% in the fifth (C5), and sixth (C6) with 66.88% removal. The photocatalyst exhibited strong magnetic responsiveness and could be quickly recovered within 30 seconds under an applied magnetic field using a common magnetic bar, achieving an average FeT recovery efficiency of 94 ± 2% per cycle. The catalyst maintained a high level of activity, with degradation efficiency dropping gradually from 84.51% in the first cycle to 66.88% after the sixth reuse, showing outstanding photocatalytic stability. The decline in efficiency suggests photocatalyst deactivation over repeated use, potentially due to improper washing after the first cycle, surface fouling from accumulated organic intermediates or persistent dye residues reducing active sites; agglomeration of 0.025FeT3 nanoparticles limiting surface area. This slight decrease in performance can be attributed to surface fouling by adsorbed dye intermediates or catalyst loss during recovery. Post-cycle FTIR analysis (Fig 10(a)) showed the preservation of the Ti-O-Ti and Fe-O bonding frameworks, while TEM micrographs in Fig. 10 (c1 and c2) revealed that the NCs retained their morphology after six reuses with no noticeable aggregation or structural deformation. These findings support the FeT’s durability and reusability in multiple dye degradation applications.

### 3.5. Photocatalytic performance prediction using machine learning approaches

#### 3.5.1. Descriptive analysis.

To analyze the photocatalytic activity for pollutant degradation, data from batch reaction experiments were collected for selected input parameters shown in S10 Table) (FeT contents (mol%), FeT dose (g/ L), reaction time (RT in minutes), and output parameters (RY145% removal). Descriptive statistical analysis shows the distribution of patterns with input features that affect the removal of pollutants. The mean FeT content (mol%) was 0.7 mol%, and it exhibited a notable positive skewness of 2.3. This indicates that most values were below 0.5 mol%, with a few high-concentration outliers appearing occasionally. The reaction time had a more uniform distribution, averaging 35 minutes. In contrast, the FeT dose had a mean of 1.1 g/L and showed a slight right skew. When it comes to FeT content (mol%), the removal affected efficiency (the average is 64.3%), but the distribution is negatively skewed (−0.9) with a median of 70.3%, indicating that the treatment generally performed well. These descriptive findings lay a solid foundation for further modeling and help understand low sensitivity parameters in future analysis (Table 2).

**Table 2:** Descriptive Statistics.

*Parameter/ Description*	N total	Mean	Standard Deviation	Variance	Skewness	Kurtosis	Minimum	1st Quartile (Q1)	Median	3rd Quartile (Q3)	Maximum
FeT contents (mol%),	270	0.74	1.5	2.36	2.30	3.64	0.01	0.025	0.05	0.5	5
Reaction time (min)	270	35	17.1	292.75	0	−1.26	10	20	35	50	60
FeT dose (g/L)	270	1.05	0.6	0.41	0.20	−1.38	0.25	0.5	1	1.5	2
RY145 removal (%)	270	64.32	18.9	360.51	−0.85	−0.33	21.09	53.79	70.29	78.95	93.62

#### 3.5.2. The correlation data of parameters.

**Fig 11:**
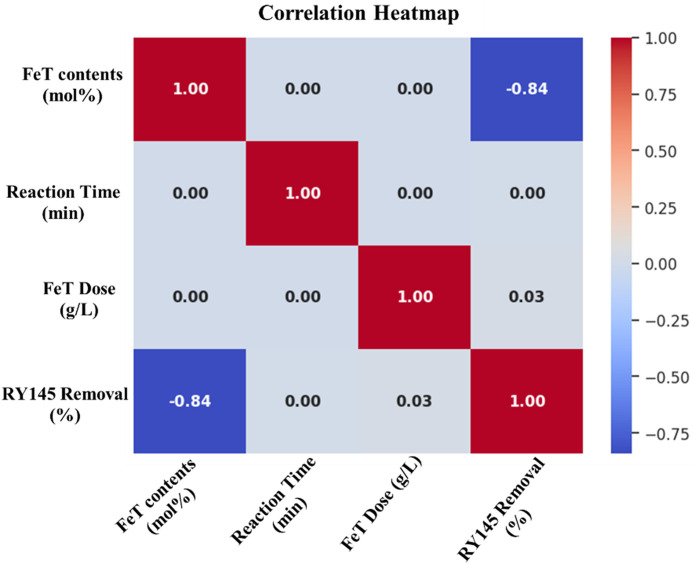
Heatmap showing correlation of parameters.

The heatmap of the correlation matrix highlights the unique relationship between variables that are essential for grasping the dynamics of RY145 removal (Fig. 11). The correlation matrix indicates that FeT content (mol%) has a strong negative correlation (−0.84) with removal efficiency, suggesting that increased FeT concentrations can hinder dye removal due to the saturation of active sites. Conversely, RT shows a negligible correlation (0.03) with the target, indicating minimal direct dependence within the tested range. The manuscript has been revised to correct this statement and emphasize the importance of optimizing FeT loading and differentiating the effects of FeT and RT for precise predictive modeling.

#### 3.5.3. Statistical indicators resulting from model performance.

Table 3 compares the performance of four neural network models on a predictive task by reporting their coefficient of determination RMSE and MAE for both training and testing datasets. All models show strong predictive accuracy with high R^2^ values and demonstrate close alignment between training and testing performance, indicating minimal overfitting. Among the models, the RNN achieves the best test performance with the test R^2^ (0.885) and lower RMSE (6.58), while CNN records the lowest MAE (4.96). Conversely, ANN performs least favorably on the test set with the R^2^ (8.77) and higher error (RMSE: 6.814, MAE: 5.334). These results suggest that the RNN architecture is the most suitable for accurately modelling the system, whereas the CNN is the least effective. The predictive capacity of these deep learning models, even though with many error indicators, shows more efficient predictive power over supervised or simple learners or tree models.

**Table 3:** Statistical metrics, indicators, and performance evaluation.

Models		R^2^	RMSE	MAE
ANN	Train	0.905	5.787	4.391
	Test	0.877	6.790	5.080
FNN	Train	0.908	5.712	4.262
	Test	0.878	6.762	5.104
CNN	Train	0.906	5.790	4.42
	Test	0.876	6.820	5.030
RNN	Train	0.907	5.730	4.320
	Test	0.880	6.700	5.060

#### 3.5.4. Regression analysis of model performance.

**Fig 12:**
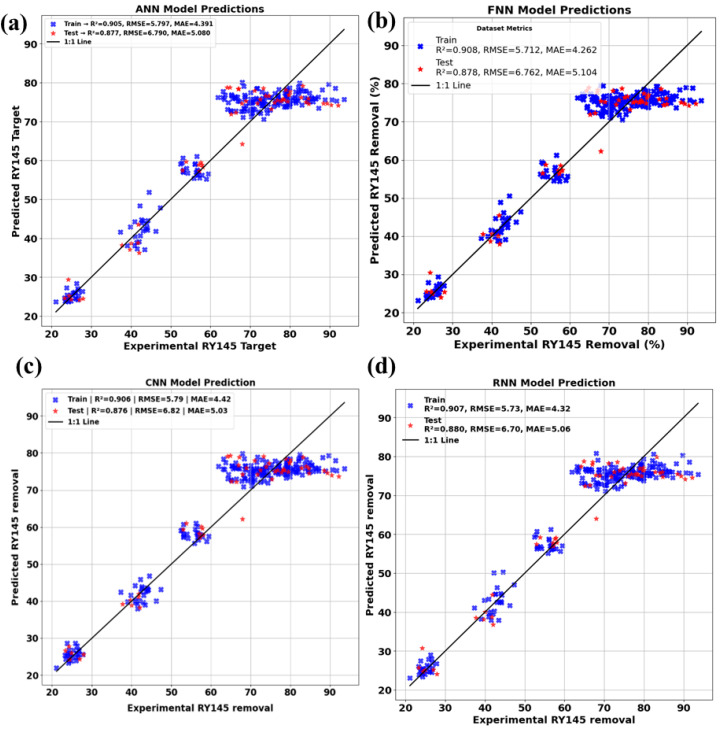
(a) ANN, (b) FNN, (c) CNN, and (d) RNN deep learning model.

The scatter plots in Fig. 12 (a-d) displayed regression plots, a common diagnostic in predictive modelling that offers a clear visual evaluation of model quality and bias by plotting projected versus actual values against a 1 ideal fit line. With a systematic scattering of data points, especially at higher experimental values, the ANN plot (Fig. 12a) showed a closer cluster of data points along the diagonal fit line, indicating better agreement between predictions and experimental results. The consistency with its higher test RMSE and MAE suggests a less accurate prediction. The plot for the FNN (Fig 12b), CNN (Fig 12c) shows the most noticeable departure from their metrics indicators than the rest of model, pointing CNN as the least performance model. The RNN plot (Fig 12d) showed the best combination of terms of capturing the nonlinear interactions between input parameters (FeT loading, reaction time, FeT dose) and RY145 removal effectiveness, as seen from it R^2^ and low error metrics on the test set quantititatively supports its status as the most reliable and impartial predictor with the most concentrated and symmetrical distribution of points around the ideal line of unity. The results validate that deep-learning models, in particular RNN, offer a significant improvement over conventional manual optimization practices by offering a dependable and accurate data-driven tool for photodegradation outcome prediction.

#### 3.5.5. 3D Surface plots analysis for model’s parameters visualization.

**Fig 13:**
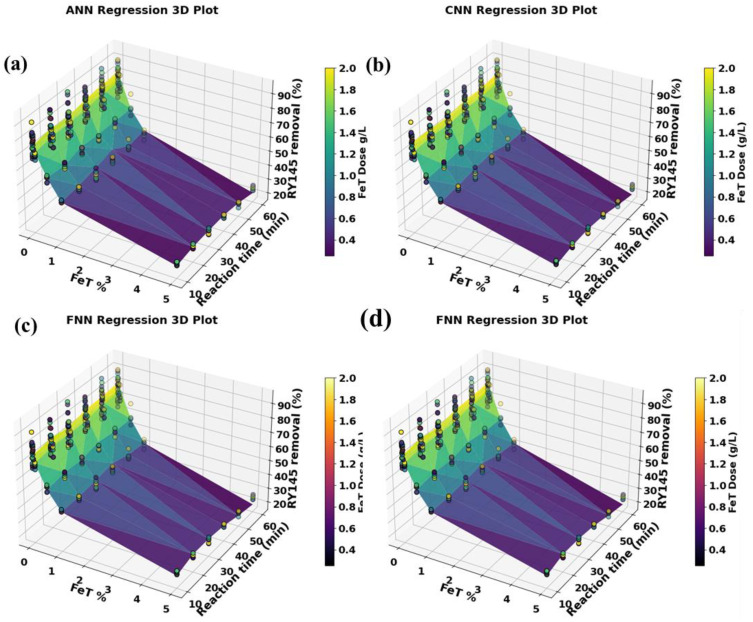
3D surface plots for parameter interactions and performance.

The 3-dimensional surface plots in Fig 13 show model-specific behaviors in capturing the underlying process dynamics by highlighting the unique response landscapes that each model learnt to predict RY145 removal as a function of FeT %, reaction and FeT dose (g/L). The ANN produces a surface with localized peaks and prominent, somewhat angular contours, indicating that it may underfit particular experimental interaction FeT %, reaction and FeT dose (g/L) against the target (RY145), producing less smooth interpolation throughout the feature space (Fig 13a). While the CNN surface exhibits slightly greater intricacy, suggesting its ability to catch tiny spatial patterns in the data input (Fig 13b), the FNN (Fig 13c) and CNN produce more continuous and gradually undulating surfaces, indicating a smoother, more generalized knowledge of the variable interactions. The RNN surface, on the other hand, is the most physicochemically plausible (Fig 13d). It is characterized by a monotonic and highly regular increase in removal efficiency with both increasing FeT %, reaction time and FeT dose. This smooth, intuitive progression without erratic fluctuations shows the RNN superior ability to model the basic sequential cause and effect relationships inherent to the reaction kinetics, aligning best with expected scientific behavior.

#### 3.5.6. Multi-metric and Taylor plot Performance validation.

**Fig 14:**
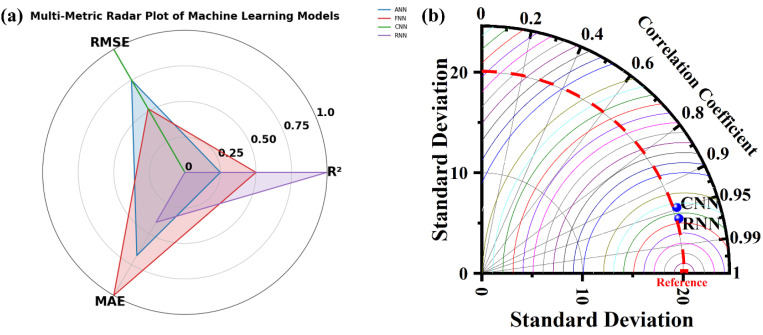
(a) Multi-metric and (b)Taylor diagram for model validation performance.

The radar plot synthesizes the multi-dimensional test performance of the four models, revealing that RNN occupies the largest area closest to the plot’s optimal perimeter, where R^2^ is maximized and RMSE and MAE are minimized with respect to other models, making it the most robust and comprehensively accurate model. The CNN exhibits the smallest area being noticeably resessed along the RMSE and MAE axes, which quantifies its highest error magnitude and lower prediction precision. A clear, comprehensive ranking of model efficacy for the given task is provided by the compact overlapping areas of the FNN and CNN, which shows that they deliver very similar intermediate performance, both superior to the CNN but falling short of RNN integrated effectiveness (Fig 14a). The Taylor diagram in Fig 14 shows a neat statistical snapshot of the best performance model prediction by showing the data correlation coefficient, standard deviation, and the RMSE compared to the reference line data. The closeness of the RNN model as the best model to the reference line has a correlation of 0.95 and a standard deviation of 10, close to that of the observed data. This close alignment reinforces the model’s strength and reliability in capturing the essential patterns of the degradation process, proving it to be a solid choice for environmental prediction tasks (Fig 14b).

#### 3.5.7. Predictive strength analysis.

**Fig 15:**
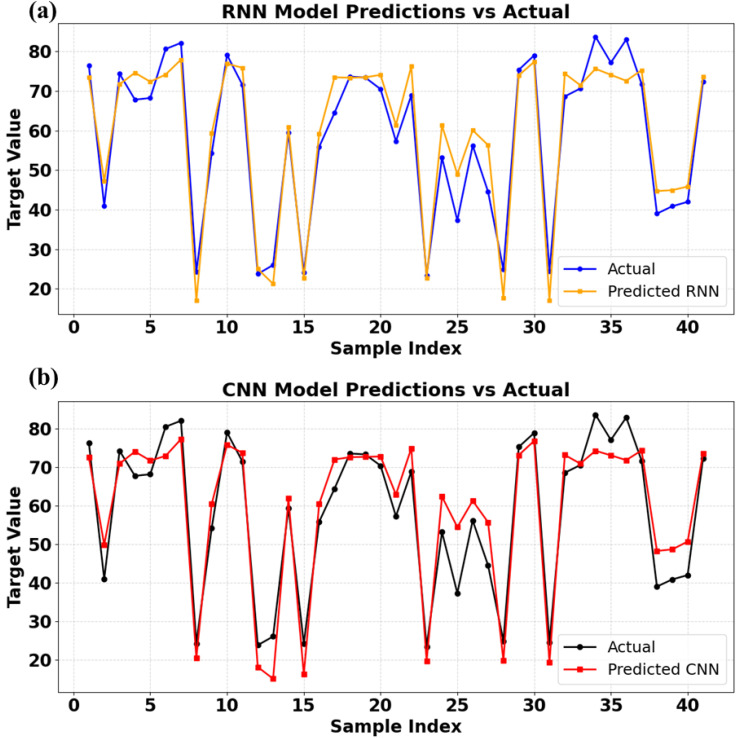
Comparative Predictive Strength Analysis. **(a)** ANN Model represents the optimal configuration, demonstrating the highest predictive accuracy and minimal residual variance. **(b)** RNN represents the baseline or underperforming model.

The quantitative superiority of the RNN over CNN as shown in Fig 15, is indicative of the difference in their performance metrics, measures, and is visually supported by the prediction plots that go with it. The CNN prediction plot (Fig 15a) shows higher test errors, which indicate a systematic under-prediction and quantitatively reflect the observable, consistent gap between the projected and actual value lines across numerous sample indices. The RNN plot (Fig 15b), on the other hand, shows a much closer visual congruence between the actual and predicted trajectories, with the two lines often overlapping. This superior temporal pattern learning translates to the RNN higher explanatory power with R^2^ = 0.880. According to this comparative analysis, the recurrent architecture of the RNN is better than the typical feedforward CNN at capturing the sequential dependencies present in the dataset, producing predictions that are more accurate and dependable.

#### 3.5.8. SHAP features impact and importance.

**Fig 16:**
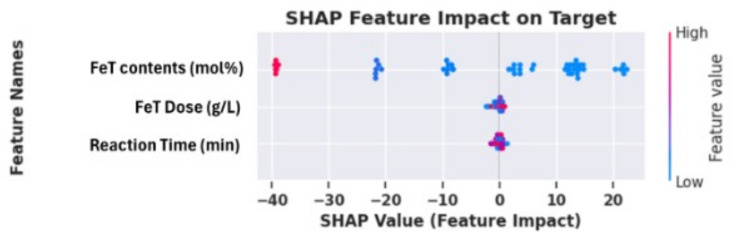
SHAP-based feature importance and impact analysis.

Complementing the regression analysis, the SHAP (SHarpley Additive exPlanations) summary plot in Fig 16 provides insight into feature-level contributions to model reductions. The plot reveals that high IFeC negatively impacts removal efficiency, while increased AD and RT positively influence the model output. This directional attribution aligns with experimental expectations and reinforces the interpretability of the model. The use of SHAP enhances transparency in models’ decision-making, a critical requirement for deploying ML models in environmental remediation contexts.

This study evaluates four deep learning models, ANN, FNN, CNN, and RNN, for predicting the photodegradation efficiency of RY145 using FeT nanocomposites with FeT% content, FeT dose, and reaction time as input variables. Among them, RNN achieved the best predictive performance on the test dataset (R2 = = 0.880, MAE = 6.7, RMSE = 5.06), followed by FNN. ANN and least CNN exhibiting least generalizations with high error metrics, likely to be due to error accumulation in tabular data. SHAP analysis identified FeT% content as the primary negative factor at higher levels, whereas FeT dose and reaction time contribute mildly but positively, reflecting experimental saturation effects and facilitating identification of optimal conditions, such as low FeT% content, for type-II heterojunction stability. Validating through Taylor diagram confirms RNN robustness (correlation = 0.95), highlighting ML advantage over manual optimization for nonlinear photocatalytic processes. The validation of this study used deep learning models (S11 Fig) to predict photocatalytic performance, based on experimental photocatalytic data, rather than the typical ML approaches for dye degradation that rely on ensemble models for tabular data. RNN model achieved R^2^ = 0.880, slightly outperforming the FNN, and CNN, but remaining specialized ensemble models such as ensemble learning tree (ELT), which are optimized for dye degradation rate constants using genetic algorithm and particle swarm optimization (GA/GSO), and emphasized factors like bandgap and reaction time [75]. Studies on the nanozeolite-RhB system achieved a higher performance (R^2^ = 0.98) through response surface methodology, underscoring the importance of architectural optimization [76].

### 3.6. Techno-economic assessment

A preliminary techno-economic evaluation was performed to assess the practical viability of the optimized 0.025FeT3 nanocomposite for treating wastewater contaminated with RY145. The estimated total operational expense (presented in Table 4) for treating one cubic meter of wastewater is around US$ 8.05 m ^‒3^ , which is competitive within the usual cost range observed for advanced oxidation processes (AOPs). The cost breakdown indicates that the process is capital-intensive regarding the catalyst, with catalyst costs being the major portion of the operational expenditure. With an optimal catalyst loading of 1 g L^‒1^ (1000 g m ^‒3^) and an estimated synthesis cost of US$ 0.08 g^‒1^, the catalyst expense amounts to about US$ 8.00 m ^‒3^, making up over 99% of the total operational cost. In contrast, the energy requirement for visible-light irradiation (0.5 kWh m ^‒3^) contributes only approximately US$ 0.045 m ^‒3^. The relatively low energy expense underscores a key advantage of the developed system, as the photocatalytic process operates effectively under visible light, eliminating the need for energy-demanding UV sources or additional oxidants like H_2_O_2_ or O_3_ often utilized in traditional AOPs.

**Table 4:** Preliminary Techno-Economic Assessment for Wastewater Treatment using 0.025FeT3 NCs.

Parameter	Value	Detail of calculation
**Technical Performance**		
Optimal Catalyst Loading (g/L)	1.00	Equivalent to 1000 g per m³ of wastewater.
Treatment Time (hour)	1.00	Time required to achieve 84.51% degradation under visible light.
Catalyst Reuse Cycles (cycles)	5.00	Number of effective uses before significant activity loss.
Energy Consumption (kWh/m³)	0.50	Based on a 500 W halogen lamp operating for 1 hour.
Economic Inputs		
Catalyst Synthesis Cost ($/g)	0.08	Estimated cost based on precursor prices and synthesis method.
Electricity Price ($/kWh)	0.12	Average industrial electricity rate.
Cost Breakdown		
A. Catalyst Cost ($/m³)	8.00	(0.08 $/g/ 5 cycles) * 1000 g/m^³^
B. Energy Cost ($/m³)	0.045	0.5 kWh/m³ * 0.09 $/kWh
**Total Operational Cost ($/m³)**	**8.045**	**A + B**

A sensitivity analysis reveals that the total treatment cost is greatly influenced by the stability and reusability of the catalyst. Currently, the catalyst retains significant activity for six reuse cycles, and the economic assessment prudently considers five effective cycles. If the catalyst’s lifespan is increased to ten cycles, the catalyst cost would drop to around US$ 4.00 m³, thereby lowering the overall treatment cost to roughly US$ 4.05 m³. This analysis illustrates that enhancing catalyst durability has a much greater effect on economic viability than variations in energy consumption. Additionally, the projected synthesis cost of US$ 0.08 g^‒1^ is based on laboratory-scale reagent prices; large-scale production is anticipated to considerably lower this cost through bulk procurement of precursors, improved synthesis methods, and possible green synthesis approaches, further enhancing economic viability. The magnetic separability of the 0.025FeT3 nanocomposite also boosts process economics by allowing for swift catalyst recovery without the need for energy-intensive filtration or centrifugation.

When compared to traditional treatment methods, using activated carbon for adsorption may present lower direct operational expenses (around US$ 0.10–0.50 m ^‒3^) [3], but it mainly shifts pollutants from water to a solid adsorbent, resulting in further challenges related to regeneration and disposal. On the other hand, photocatalysis allows for the complete breakdown of organic pollutants, making it a more environmentally sustainable treatment option. However, many existing photocatalytic systems depend on costly noble metals or intricate manufacturing processes (for example, Ag-Zn-BTC/GO, Ag-based metal–organic framework composites) [17], which can substantially raise operational expenses. Conventional AOPs such as ozone- or UV-based treatments typically range between US$ 0.50 and 15.00/m^3^ depending on pollutant load and energy rates [11,14]. In this regard, the 0.025FeT3 system offers an advantageous blend of material cost, ease of operation, and catalytic effectiveness, underscoring its potential as a viable photocatalyst for treating dye-laden wastewater.

### 3.7. Potential Environmental Concerns of Fe_3_O_4_/TiO_2_ NCs

Although 0.025FeT3 nanocomposites exhibit excellent performance in pollutant removal and offer the advantage of magnetic separation, several environmental concerns must be considered for their safe and sustainable application in real-world wastewater treatment systems. One key concern is the potential leaching or release of nanoparticles into the environment during or after the treatment process. Incomplete magnetic recovery, aggregation, or aging of the nanocomposites can lead to their accumulation in aquatic ecosystems, where they may pose ecotoxicological risks to aquatic organisms and disrupt microbial communities. Furthermore, the long-term stability and fate of 0.025FeT3 nanocomposites in complex wastewater matrices remain uncertain. Factors such as pH fluctuations, ionic strength, and the presence of natural organic matter may influence the dispersion and transformation of nanoparticles. As highlighted in the recent study by [77], oxidative stress and membrane damage in aquatic biota can occur due to exposure to TiO_2_-based nanomaterials, raising concerns about their environmental safety. Iron(II, III) oxide/titanium dioxide (Fe_3_O_4_/TiO_2_) NCs show minimal iron (Fe) and titanium (Ti) leaching, with concentrations typically below 0.2 mg L^−1^, according to reports [78]. This is much lower than the World Health Organization’s (WHO) aesthetic-based standards for drinking water of 0.3 mg/L for Fe and 0.5 mg/L for Ti, proving the material’s safety for water treatment applications. HPLC findings indicate stepwise azo cleavage and oxidative mineralization of RY145 by FeT under visible light; further identification of intermediates and toxicity assessment should be conducted using LC-MS/GC-MS and ecotoxicity assays.

## 4. Conclusions

This study shows that designing a mesoporous Fe_3_O_4_/TiO_2_ nanocomposite with controlled interfacial contacts improves photocatalytic activity under visible-light irradiation. The designed system combines effective charge separation, extended light absorption, mesoporosity-driven mass transfer, and magnetic recoverability to provide a viable and scalable solution for wastewater treatment. The findings clearly demonstrate that interfacial engineering, rather than simple compositional alteration, is a critical element influencing photocatalytic efficiency and application. This research investigated the structural, optical, morphological, magnetic, and photocatalytic properties of magnetite/TiO_2_ nanocomposite (Fe_3_O_4_/TiO_2_ NCs) used to decolorize RY145 dye under visible light. RY145 is chosen as a representative pollutant given its industrial origin at Kohinoor Textile Mills, ensuring practical relevance and closing the gap between laboratory studies and real-world wastewater applications. The findings illustrate how adding Fe_3_O_4_ to TiO_2_ improves carrier separation, reduces electron-hole recombination, and improves photocatalytic efficiency by promoting electron transfer. The 0.025FeT3 NCs had the highest dye removal efficiency (84.51% %) under visible light at neutral pH, with photocatalytic degradation driven predominantly by holes and hydroxyl radicals. Simultaneous adsorption and photocatalysis were indicated by the pseudo-first-order kinetic model of degradation and the Langmuir-Hinshelwood isotherm, which dominated the process. PL data showed that photogenerated electron-hole pairs had higher separation efficiency and greater photocatalytic activity. XRD findings demonstrate stable anatase TiO_2_ up to 500 °C, whereas the strength of the Fe-O and Ti-O peaks increases with higher calcination (0.025FeT4), indicating improved crystallinity and ferromagnetic behavior. The trend of saturation magnetization (Ms) (FeT5)> Ms(FeT3)> Ms(FeT4) is likely influenced by the calcination temperature. With negligible Hc and Mr, the behavior is more accurately described as superparamagnetic or soft ferromagnetic, depending on the hysteresis loop shape and particle size. Scavenger studies provide indirect evidence for •OH or h^+^ radicals, requiring further verification using techniques like ESR or fluorescence spectroscopy. Recycling studies, supported by FTIR and TEM analyses, confirmed that the 0.025FeT3 retained its structural integrity and high photocatalytic efficiency after multiple reuse cycles, validating its durability for sustainable dye degradation applications. HPLC chromatogram validated effective photocatalytic degradation of RY145 via azo bond cleavage and mineralization, confirming FeT efficiency under visible light. The optimized Fe_3_O_4_ (~0.025 mol%) and moderated calcination (~300 °C) engineered a stable type-II Fe_3_O_4_/TiO_2_ heterojunction with efficient charge separation, preserved mesoporosity, and soft magnetism. This enabled neutral-pH visible-light degradation at competitive EEO with rapid magnetic recovery and minimal leaching. The evaluation of deep learning algorithms shows that Artificial Neural Networks (ANN) and Feedforward Neural Networks (FNN) provides the highest predictive accuracy for RY145%removal, demonstrating superior R² values compared to Convolutional Neural Networks (CNN) and Recurrent Neural Networks (RNN) indicating these models effectively capture the complex, nonlinear relationships and interactions among Influential Factors (IFeC), Reaction Time (RT), and Adsorption Duration (AD), allowing for robust generalization across different experimental conditions. It was confirmed from both SHAP (SHapley Additive exPlanations) and Taylor analysis that IFeC is the most significant parameter, while RT and AD positively contribute to the efficiency of RY145 removal in the best-performing models. These findings establish NN frameworks as powerful tools for optimizing photocatalytic processes and enhancing data-driven reactor design. This research identifies various limitations, such as the absence of detailed LC–MS analysis for quantification of degradation by-products, the lack of practical wastewater testing and ESR validation for reactive species, and limited assessment at the field scale, which need to be performed in future studies.

## Supporting information

S1 Table Removal efficiency and reusability of Fe_3_O_4_/TiO_2_ photocatalyst. (PDF)

S2 Fig VOSviewer bibliometric map (2015–2025) on Fe_3_O_4_/TiO_2_ composites and Reactive Yellow 145 removal. (TIF)

S3 Table List of chemicals and materials used with estimated cost. (PDF)

S4 Table Comparison of the 4 models’ test predictive performance. (PDF)

S5 Fig The network architecture for the 4 models. (TIF)

S6 Fig Thermogravimetric analysis (TGA) of the uncalcined 0.025FeT nanocomposites. (TIF)

S7 Fig Adsorption equilibrium data for Reactive Yellow 145 using (a) Langmuir and the (b) Freundlich isotherm model on 0.025FeT-3 NCs. (TIF)

S8 Fig Effect of substrate. (TIF)

S9 Fig HPLC analysis of degradation by-products using 0.025FeT3 (60 min). (TIF)

S10 Table Data collection. (PDF)

S11 Fig ML Model Performance Analysis by MAE/Epochs. (TIF)

S12 Fig EDS image. (TIF)
