# Supplementary material for: Synergistic interfacial engineering of mesoporous magnetic metal oxide TiO2 nanocomposites for sustainable visible-light photocatalysis: Experimental insights and ML-based performance prediction
Source: PLoS One. 2026 Jun 2;21(6):e0348881. doi: 10.1371/journal.pone.0348881 (PMC13229325; doi:10.1371/journal.pone.0348881)
Supplement: S1 Table — (PDF) [file pone.0348881.s001.pdf]

S1 Table. Removal efficiency and reusability of Fe_3O_4/TiO_2 photocatalyst.

S. No	Pollutant	Synthesis Method	Removal Efficiency (%) Initial	Removal Efficiency (%) Final	Light Source	Reuse	Photocatalyst Dose mg/L	pH	References
1	MB	CP-SG-CD	90	-	UV Vis	1	10	5- 7	[1]
2	MO	SG	99.68	-	Solar	1	20	5	[2]
3	R6G	CP	98.47	97.3	UV	5	40	3,7,10	[3]
4	MB	SV	97.56	89.9	UV Vis	5	50	7	[4]
5	Ciprox	MV	94	91	UV-A	3	50	6.5	[5]
6	RhB	HT	91	89	Solar	5	-	-	[6]
7	MB	HT	84	82	Solar	3	-	-	[7]
8	MB	SV-US	89.2	86.4	-	2	40	3	[8]
9	MB	C	91.16	-	Ads	-	40	8	[9]
10	Cd (II)		98.24	72.09	-	5	-	> 7	[10]
11	RhB	SG	98.12	90	UV	5	1		[11]
12	MB	SG	62.67				1		[12]
13	AMX	CP	99.9	89	UV-C	5	600	3-11	[13]
14	**RY145**	CP-SG	**84.51**	**68**	**Vis**	**5**	**60**	**7**	**This study**

NR(Natural Red); RhB (Rhodamine B); Rhd 6G (Rhodamine 6G); Ciprox (Ciprofloxacin); MB (Methylene Blue); MO (Methyl Orange), AMX (Amoxicillin); RY145 (Reactive Yellow 145), SV (Solvothermal method), MV(microwave-assisted method), CP-SG (Co-Precipitation-assisted Sol-gel method), Ads (adsorption), CP-Imp (co precipitation–impregnation method), HT (Hydrothermal method), SV-US (Solvothermal Ultrasonic-Assisted method), CP-SG-CD (Co-Precipitation, Sol-Gel and Chemical Deposition methods)

References

1. Tedsree K, Temnuch N, Sriplai N, Pinitsoontorn S. Ag modified Fe_3O_4@TiO_2 magnetic core-shell nanocomposites for photocatalytic degradation of methylene blue. Materials Today: Proceedings. 2017;4(5, Part 2):6576-84. doi: https://doi.org/10.1016/j.matpr.2017.06.170.
2. Gebrezgiabher M, Gebreslassie G, Gebretsadik T, Yeabyo G, Elemo F, Bayeh Y, et al. A C-Doped TiO_2/Fe_3O_4 Nanocomposite for Photocatalytic Dye Degradation under Natural Sunlight Irradiation. Journal of Composites Science. 2019;3(3):75. doi: https://doi.org/10.3390/jcs3030075.
3. Xie E, Zheng L, Li X, Wang Y, Dou J, Ding A, et al. One-step synthesis of magnetic-TiO_2-nanocomposites with high iron oxide-composing ratio for photocatalysis of rhodamine 6G. PLOS ONE. 2019;14(8):e0221221. doi: 10.1371/journal.pone.0221221.
4. Zhang Q, Yu L, Xu C, Zhang W, Chen M, Xu Q, et al. A novel method for facile preparation of recoverable Fe_3O_4@TiO_2 core-shell nanospheres and their advanced photocatalytic application. Chemical Physics Letters. 2020;761:138073. doi: https://doi.org/10.1016/j.cplett.2020.138073.
5. Gabelica I, Ćurković L, Mandić V, Panžić I, Ljubas D, Zadro K. Rapid Microwave-Assisted Synthesis of $Fe_3O_4/SiO_2/TiO_2$ Core-2-Layer-Shell Nanocomposite for Photocatalytic Degradation of Ciprofloxacin. Catalysts. 2021;11(10):1136. PubMed PMID: doi:10.3390/catal11101136.

6. Madima N, Kefeni KK, Mishra SB, Mishra AK, Kuvarega AT. Fabrication of magnetic recoverable Fe_3O_4/TiO_2 heterostructure for photocatalytic degradation of rhodamine B dye. Inorganic Chemistry Communications. 2022;145:109966. doi: https://doi.org/10.1016/j.inoche.2022.109966.
7. Shilpa G, Kumar PM, Deepthi PR, Sukhdev A, Bhaskar P, Kumar DK. Improved Photocatalytic Performance of Fe_3O_4/TiO_2 Thin Film in the Degradation of MB Dye Under Sunlight Radiation. Brazilian Journal of Physics. 2023;53(2):38. doi: 10.1007/s13538-022-01243-z.
8. Halfadji A, Naous M, nadia Kharroubi K, el zahraà Belmehdi F, Rajendrachari S. An ultrasonic-assisted Synthesis, Characterization, and application of Nano-Fe_3O_4/TiO_2 as nano-catalyst for the removal of organic dye by Like-Photo-Fenton reactions. Inorganic Chemistry Communications. 2023;158:111686. doi: https://doi.org/10.1016/j.inoche.2023.111686.
9. Almutairi ST. Fabrication and catalytic activity of TiO_2/Fe_3O_4 and Fe_3O_4/β-cyclodextrin nanocatalysts for safe treatment of industrial wastewater. Heliyon. 2024;10(15):e35400. doi: https://doi.org/10.1016/j.heliyon.2024.e35400.
10. Chander S, Yadav S, Sharma HR, Gupta A. Sequestration of Cd (II) utilizing biowaste-fabricated recyclable mesoporous magnetite (Fe_3O_4) nano-adsorbent: Process optimization, thermodynamic investigation, simulation modeling, and feasibility for electroplating effluent. Journal of Alloys and Compounds. 2024;986:174088. doi: https://doi.org/10.1016/j.jallcom.2024.174088.
11. Li Y, Zhang F, Xu K, Yang Z, Dong Z. The synergistic enhancement of photocatalytic activity by Fe_3O_4–TiO_2 nanosheets for rhodamine B degradation. Colloids and Surfaces A: Physicochemical and Engineering Aspects. 2024;700:134803. doi: https://doi.org/10.1016/j.colsurfa.2024.134803.
12. Rajabathar JR, Thankappan R, Sutha A, Al-Lohedan H, Mahmoud Karami A, Ashok Kumar S, et al. Enhanced photocatalytic activity of magnetite/titanate (Fe_3O_4/TiO2) nanocomposite for methylene blue dye degradation under direct sunlight. Optical Materials. 2024;148:114820. doi: https://doi.org/10.1016/j.optmat.2023.114820.
13. Moein H, Shima N, Elham N, Hossein K, and Abdipour H. Investigating the photocatalytic removal of amoxicillin from aqueous solutions by Fe-TiO_2@Fe_3O_4 magnetic nanoparticles. Characteristics/effect of parameters/kinetic study. Chemical Engineering Communications. 2025;212(7):1031-47. doi: 10.1080/00986445.2024.2445230.
