# Supplementary figures and images for: Synergistic interfacial engineering of mesoporous magnetic metal oxide TiO2 nanocomposites for sustainable visible-light photocatalysis: Experimental insights and ML-based performance prediction
Source: PLoS One. 2026 Jun 2;21(6):e0348881. doi: 10.1371/journal.pone.0348881 (PMC13229325; doi:10.1371/journal.pone.0348881)

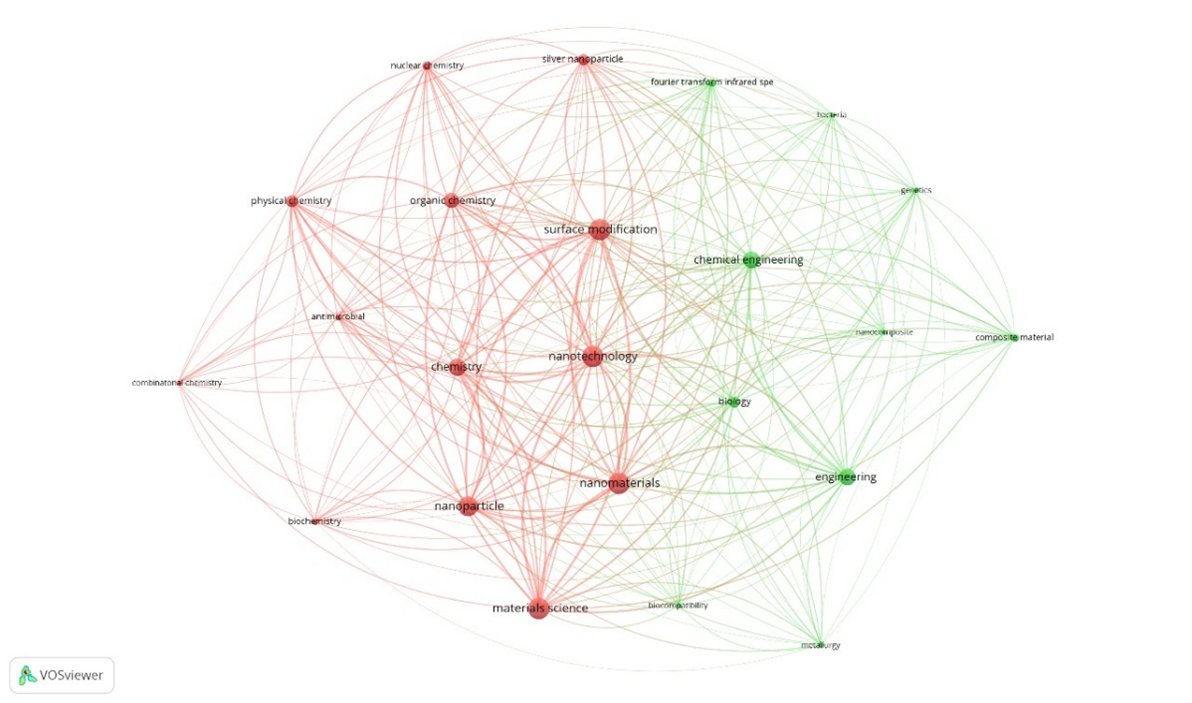

Supplement: S2 Fig — (TIF) [file pone.0348881.s002.tif]

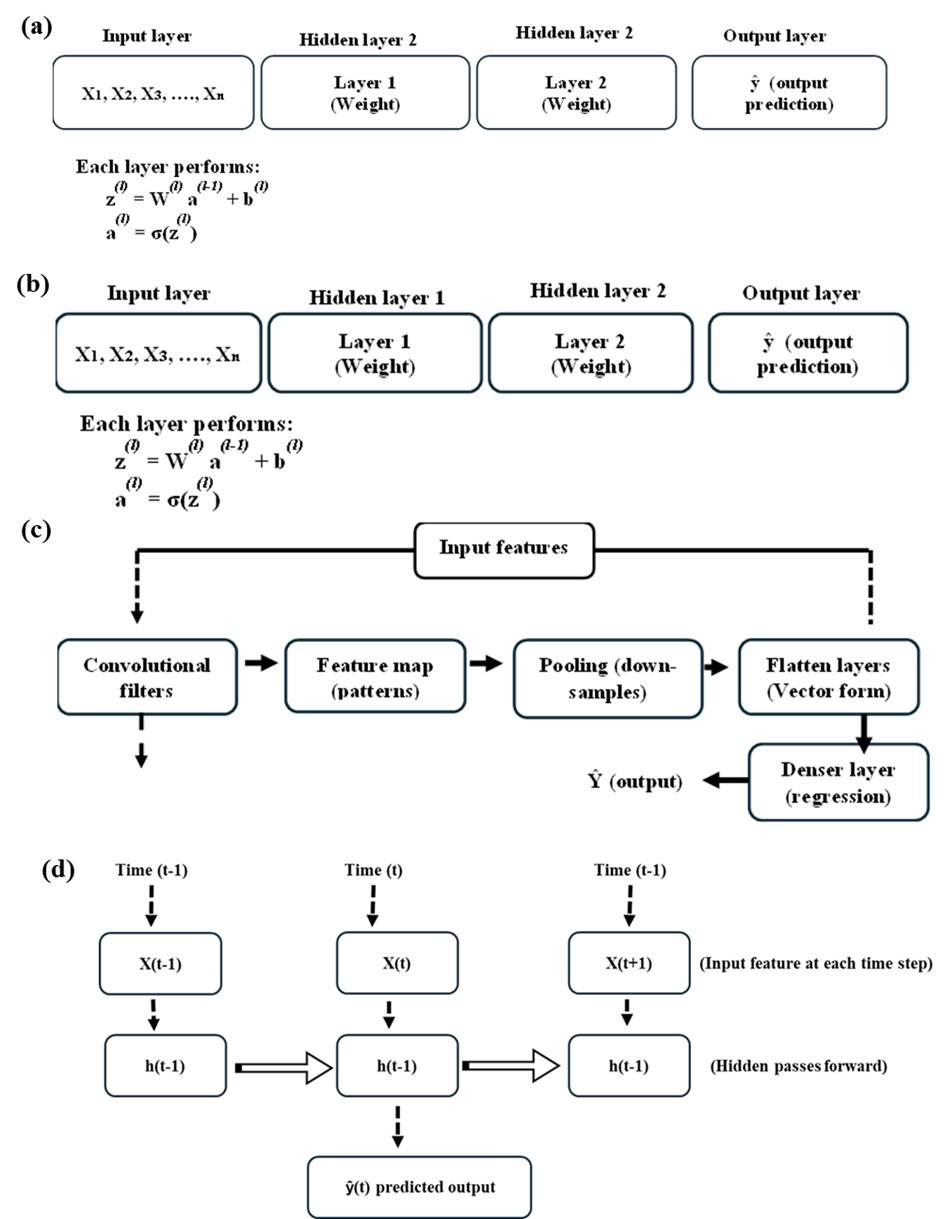

Supplement: S5 Fig — (TIF) [file pone.0348881.s005.tif]

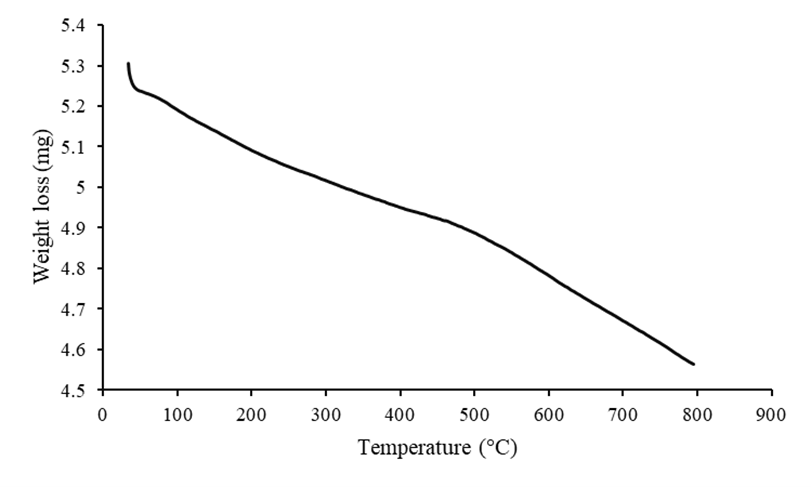

Supplement: S6 Fig — (TIF) [file pone.0348881.s006.tif]

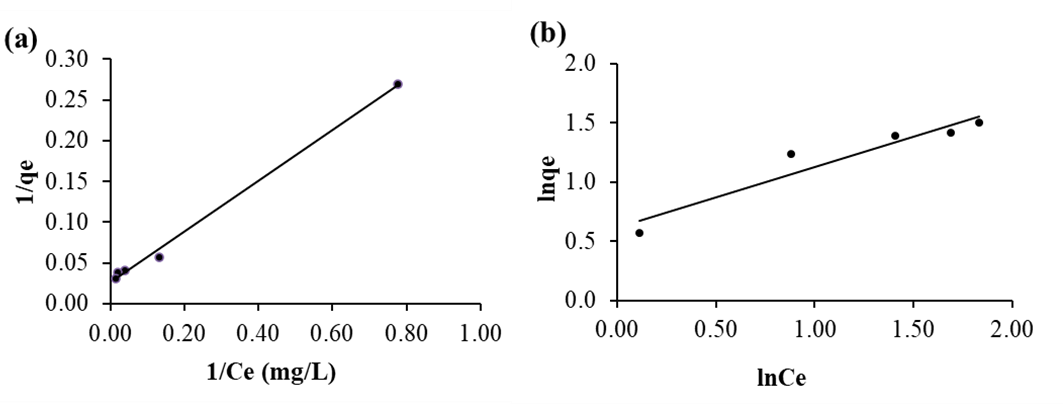

Supplement: S7 Fig — (TIF) [file pone.0348881.s007.tif]

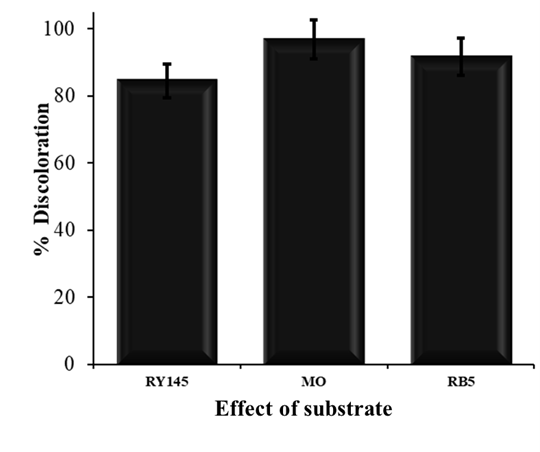

Supplement: S8 Fig — (TIF) [file pone.0348881.s008.tif]

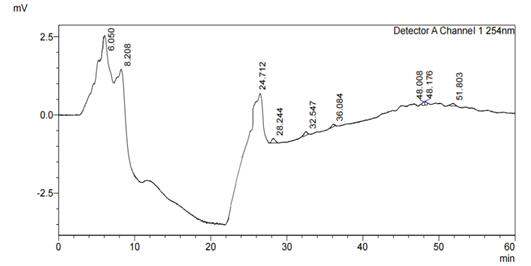

Supplement: S9 Fig — (TIF) [file pone.0348881.s009.tif]

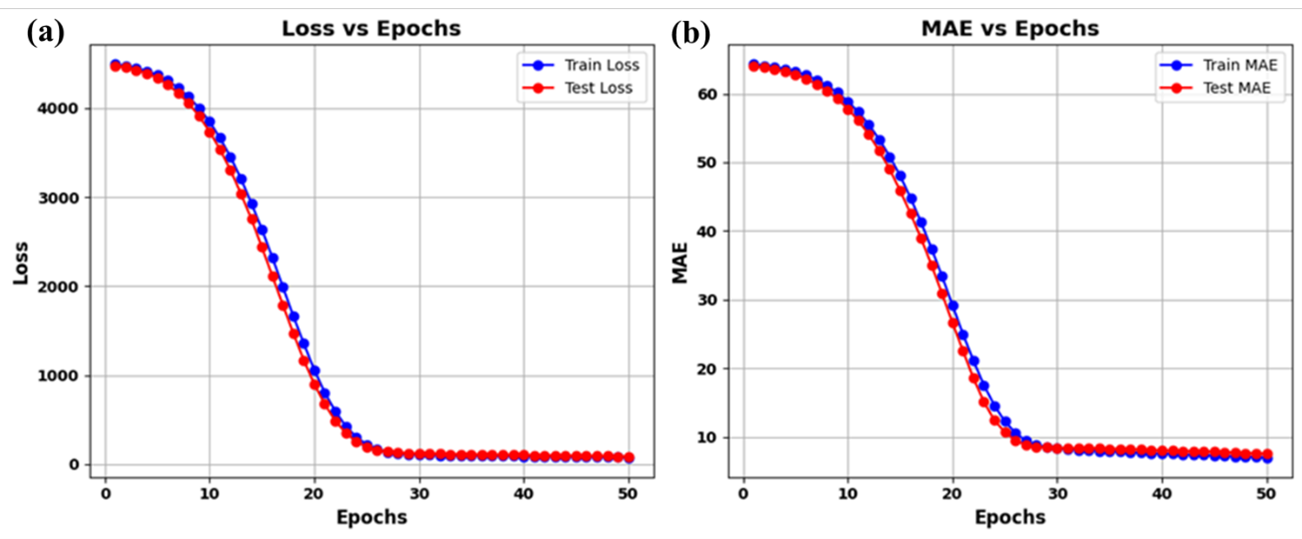

Supplement: S11 Fig — (TIF) [file pone.0348881.s011.tif]

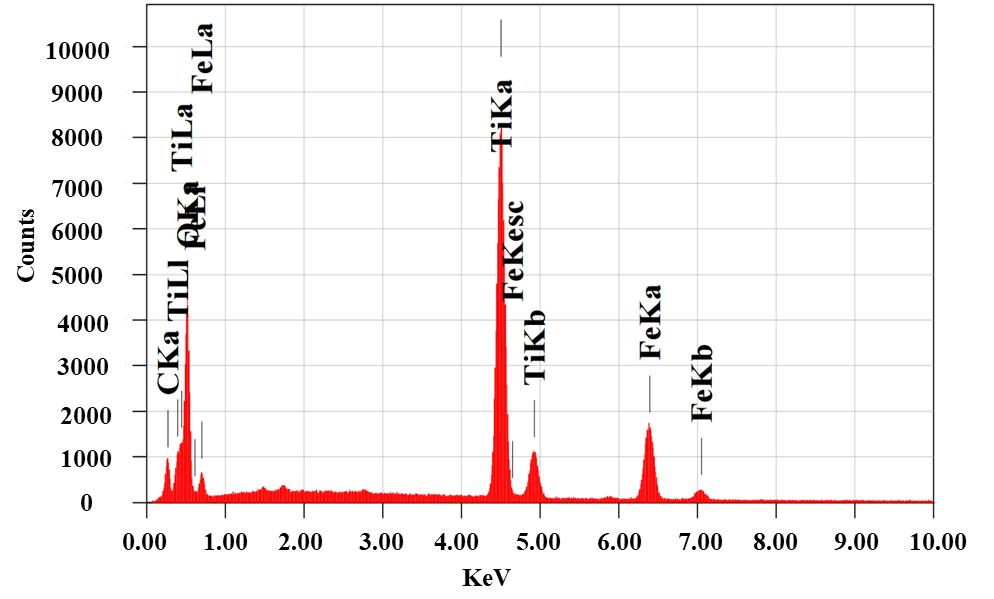

Supplement: S12 Fig — (TIF) [file pone.0348881.s012.tif]
