# Supplementary material for: Synergistic interfacial engineering of mesoporous magnetic metal oxide TiO2 nanocomposites for sustainable visible-light photocatalysis: Experimental insights and ML-based performance prediction
Source: PLoS One. 2026 Jun 2;21(6):e0348881. doi: 10.1371/journal.pone.0348881 (PMC13229325; doi:10.1371/journal.pone.0348881)
Supplement: S3 Table — (PDF) [file pone.0348881.s003.pdf]

S3 Table: List of chemicals and materials used with estimated cost

All the chemicals used in this study, along with the estimated cost and lab-scale cost estimation of the synthesized composite, are listed in Table S3:

Cost Analysis of the Chemicals							
**Sr No**	**Chemical Name**	**Company**	**Purity**	**Quantity**	**Price in US Dollars ($)**	**Price in PKR Rs**	**Price of 1 gm of NC in Rs**
**1**	Iron(II) sulfate heptahydrate ($FeSO_4.7H_2O$)	Sigma-Aldrich	98.00%	500g	139.42	39275.7	78.55
**2**	Ammonia (NH_3)	Analar Normapur	28-32%	1L	40	11268.3	11.27
**3**	Iron(III) chloride hexahydrate ($FeCl_3.6H_2O$)	Sigma-Aldrich	98.00%	500g	142.69	40196.9	80.39
**4**	Sodium carbonate (Na_2CO_3)	Sigma-Aldrich	99.00%	500g	29.41	8285.02	16.57
**5**	Copper sulphate pentahydrate ($CuSO_4.5H_2O$)	Sigma-Aldrich	98.00%	500g	126.46	35624.8	71.25
**6**	Ethylene Glycol ($HOCH_2$ CH_2OH)	Dae-Jung	99.00%	1 L	100.1	28198.9	28.2
**7**	Sodium silicate ($Na_2SiO_3.9H_2O$)	Sigma-Aldrich	98.00%	500g	6.33	1783.21	3.57
**8**	Calcium Carbonate ($CaCO_3$)	Sigma-Aldrich	99.00%	500g	92.8	26142.5	52.28
**9**	Hydrochloric acid (HCl)	Sigma-Aldrich	37%	1L	71.89	20252	20.25
**10**	Titanium(IV) isopropoxide ($Ti[OCH(CH_3)_2]_4$)	Sigma-Aldrich	97.00%	0.5L	100.1	28198.9	56.4
							**418.73**
