# Supplementary material for: Synergistic interfacial engineering of mesoporous magnetic metal oxide TiO2 nanocomposites for sustainable visible-light photocatalysis: Experimental insights and ML-based performance prediction
Source: PLoS One. 2026 Jun 2;21(6):e0348881. doi: 10.1371/journal.pone.0348881 (PMC13229325; doi:10.1371/journal.pone.0348881)
Supplement: S4 Table — (PDF) [file pone.0348881.s004.pdf]

**S4 Table. Comparison of the 4 models' test predictive performance**

| FeT % | Reaction time (min) | FeT dose (g/L) | Actual | ANN Predicted | FNN Predicted | CNN Predicted | RNN Predicted |
|---|---|---|---|---|---|---|---|
| 0.035 | 30 | 0.5 | 76.4 | 72.6 | 72.8 | 74.1 | 73.4 |
| 1 | 50 | 1.5 | 41.0 | 49.9 | 40.2 | 41.4 | 47.2 |
| 0.1 | 20 | 0.5 | 74.3 | 71.0 | 70.6 | 71.6 | 71.7 |
| 0.1 | 60 | 0.5 | 67.8 | 74.1 | 72.5 | 73.0 | 74.6 |
| 0.1 | 50 | 1 | 68.2 | 71.8 | 69.9 | 71.1 | 72.3 |
| 0.035 | 40 | 1.5 | 80.6 | 73.0 | 72.9 | 74.5 | 74.1 |
| 0.015 | 10 | 2 | 82.1 | 77.3 | 77.8 | 75.2 | 77.9 |
| 5 | 40 | 0.25 | 24.2 | 20.4 | 24.5 | 24.4 | 17.0 |
| 0.5 | 20 | 1.5 | 54.2 | 60.5 | 56.1 | 56.6 | 59.3 |
| 0.015 | 60 | 1.5 | 79.1 | 75.9 | 76.4 | 76.0 | 76.9 |
| 0.015 | 20 | 0.25 | 71.5 | 73.8 | 74.9 | 75.6 | 75.8 |
| 5 | 30 | 2 | 23.8 | 18.1 | 25.4 | 23.3 | 25.0 |
| 5 | 40 | 1.5 | 26.0 | 15.0 | 23.9 | 22.7 | 21.3 |
| 0.5 | 50 | 1.5 | 59.4 | 61.9 | 56.7 | 58.4 | 60.9 |
| 5 | 50 | 1.5 | 24.1 | 16.1 | 23.6 | 23.0 | 22.7 |
| 0.5 | 20 | 1 | 55.8 | 60.5 | 55.0 | 56.4 | 59.2 |
| 0.1 | 50 | 0.25 | 64.5 | 72.1 | 72.9 | 72.9 | 73.4 |
| 0.05 | 40 | 0.5 | 73.6 | 72.6 | 72.6 | 74.3 | 73.3 |
| 0.1 | 60 | 1 | 73.4 | 72.7 | 70.9 | 72.3 | 73.4 |
| 0.01 | 20 | 1 | 70.4 | 72.8 | 73.6 | 73.6 | 74.0 |
| 0.5 | 60 | 1 | 57.3 | 62.9 | 56.4 | 58.7 | 61.4 |
| 0.01 | 60 | 1 | 68.9 | 75.0 | 74.3 | 75.4 | 76.2 |
| 5 | 60 | 1 | 23.4 | 19.6 | 22.7 | 23.1 | 22.7 |
| 0.5 | 10 | 0.5 | 53.2 | 62.4 | 57.7 | 58.3 | 61.3 |
| 1 | 60 | 0.5 | 37.3 | 54.5 | 42.7 | 43.7 | 48.9 |
| 0.5 | 40 | 0.25 | 56.2 | 61.3 | 56.8 | 60.0 | 60.1 |
| 1 | 60 | 2 | 44.5 | 55.7 | 52.5 | 46.9 | 56.3 |
| 5 | 10 | 0.5 | 24.9 | 19.8 | 26.0 | 23.9 | 17.6 |
| 0.05 | 30 | 2 | 75.3 | 73.2 | 74.7 | 73.8 | 73.9 |
| 0.035 | 10 | 2 | 79.0 | 76.9 | 77.1 | 74.5 | 77.3 |
| 5 | 20 | 0.25 | 24.4 | 19.3 | 25.6 | 24.6 | 17.1 |
| 0.01 | 30 | 1 | 68.6 | 73.2 | 73.3 | 74.4 | 74.4 |
| 0.1 | 30 | 0.5 | 70.6 | 71.0 | 70.3 | 71.8 | 71.5 |
| 0.015 | 10 | 0.5 | 83.7 | 74.3 | 75.3 | 75.0 | 75.6 |
| 0.015 | 30 | 0.5 | 77.1 | 73.1 | 73.6 | 74.8 | 74.1 |
| 0.05 | 10 | 1 | 83.0 | 71.9 | 73.5 | 72.5 | 72.5 |
| 0.015 | 40 | 2 | 71.7 | 74.4 | 77.1 | 76.8 | 75.2 |
| 1 | 30 | 1.5 | 39.0 | 48.3 | 36.7 | 39.9 | 44.7 |
| 1 | 40 | 1.5 | 40.9 | 48.7 | 37.3 | 40.9 | 44.9 |
| 1 | 50 | 0.5 | 42.0 | 50.7 | 38.8 | 40.8 | 45.8 |
| 0.1 | 20 | 2 | 72.3 | 73.6 | 73.2 | 71.7 | 73.6 |
