# Supplementary material for: Synergistic interfacial engineering of mesoporous magnetic metal oxide TiO2 nanocomposites for sustainable visible-light photocatalysis: Experimental insights and ML-based performance prediction
Source: PLoS One. 2026 Jun 2;21(6):e0348881. doi: 10.1371/journal.pone.0348881 (PMC13229325; doi:10.1371/journal.pone.0348881)
Supplement: S10 Table — (PDF) [file pone.0348881.s010.pdf]

S10 Table Data Collection

Parameter 1		Parameter 2		Parameter 3	
**Initial iron concentration (mol%)**	**Removal (%)**	**Removal time (min)**	**Removal (%)**	**Dosage (g/L)**	**Removal (%)**
0.01	66.6	10	50.8	0.25	77.9
0.015	75.9	20	60.4	0.5	72.4
0.025	84.51	30	74.2	1	84.51
0.035	77.5	40	78.2	1.5	96.1
0.05	76.1	50	82.4	2	98.2
0.1	67.4	60	84.51	-	-
0.5	55.0	-	-	-	-
1	41.3	-	-	-	-
5	24.8	-	-	-	-

1. Machine learning Hyperparameters optimization

Model performance critically depends on the choice and configuration of hyperparameters during training. Key settings such as learning rate batch size activation function and network architecture directly influence convergence and generalization across models like ANN, FNN, CNN and RNN. The learning rate determines the magnitude of weight updates per optimization step; lower values typically yield more stable but slower convergence, while higher values risk divergence or overfitting. Batch size the number of examples processed before each update affects gradient estimation stability and generalization. Activation functions such as ReLU tanh introduce non-linearity essential for deep model expressivity. In CNNs, choices like filter number and kernel size further shape representational capacity. In RNNs, recurrent configurations and gating mechanisms modulate sequence learning. Proper hyperparameter tuning, often found as hyperparameter optimization (HPO), substantially enhances predictive accuracy and training efficiency in deep learning models, and recent systematic studies highlight the role of advanced HPO techniques in optimizing CNN, LSTM and (a form of RNN) performance [14-16]

Model	Hyperparameters
**ANN**	Hidden layer sizes: [64, 32], Hidden activation: relu, Output activation: linear, Optimizer: Adam, Initial learning rate: 0.001, Loss function: mse, Batch size: 16, Maximum epochs: 500, Early stopping: Enabled, Early stopping, patience: 20, Validation fraction: 0.2, Random state: 42 (train–test split).
**FNN**	Hidden layers:[64, 32], Activation function: relu, Output activation : linear, Optimizer: Adam, Learning rate: 0.001 , Loss function: mse, Metrics: ['mae'], Epochs

	(max): 500, Batch size: 16, Validation split : 0.2, Early stopping enabled: True, Early stopping monitor: Val-loss, Early stopping patience : 20, Random seed: 42.
**CNN**	Conv1d-2 (Conv1D) (None, 2, 64), conv1d-3 (Conv1D) (None, 1, 32), flatten_1 (Flatten) (None, 32), dense-11 (Dense) (None, 16), dense_12 (Dense) (None, 1, Optimizer: Adam, Learning rate: 0.0010000000474974513, Loss function: Mean Squared Error (MSE), Batch size: 16, Maximum epochs: 500
**RNN**	RNN Units: 64, Dense Units: 32, Activation Function: relu, Optimizer: adam, Loss Function: mse, Metrics: ['mae'], Epochs: 500, Batch Size: 16, Validation Split: 0.2, Early Stopping Monitor: val-loss, Early Stopping, Patience: 20, Restore Best Weights: True, Epoch 1/500.
